# Comparative membrane incorporation of omega-3 fish oil triglyceride preparations differing by degree of re-esterification: A sixteen-week randomized intervention trial

**DOI:** 10.1371/journal.pone.0265462

**Published:** 2023-01-27

**Authors:** Scott T. Minton, Anthony L. Almada, Joseph L. Evans, Maggie Laidlaw, Joar Opheim

**Affiliations:** 1 Department of Research, Nordic Naturals, Inc., Watsonville, California, United States of America; 2 Vitargo Global Sciences, Inc., Dana Point, California, United States of America; 3 P and N Development Ventures, Saint Louis, Missouri, United States of America; 4 Nutrasource Diagnostics Inc., Guelph, Ontario, Canada; Oregon State University, UNITED STATES

## Abstract

**Background:**

Fish oil is routinely concentrated into unmodified triglycerides, or trans-esterified into an ethyl ester form. Re-esterification of the ethyl ester form yields re-esterified triglycerides (rTG), which are reportedly more bioavailable than ethyl ester forms. However, the fidelity of the re-esterification process may yield variable triglyceride forms, with only 55–60% being rTG.

**Objective:**

To determine whether the blood lipidomic response to supplementation with two rTG supplements, varying by degree of re-esterification, would differ between treatments.

**Design:**

This was a double-blind, parallel-design, single-center, 128-day study with sixty young, healthy subjects randomized into two groups. One group received a >95% rTG (Ultimate Omega®), as 1,000 mg capsules containing 325 mg eicosapentaenoic acid (EPA) and 225 mg docosahexaenoic acid (DHA), and the other received a <70% rTG (MEG-3) as 1,000 mg capsules containing 300 mg EPA and 200 mg DHA. Total intake was 2,750 and 2,500 mg EPA+DHA for the Ultimate Omega® and MEG-3 groups, respectively, with blood drawn at 4, 16 and 24 weeks and analyzed for serum and erythrocyte phospholipid fatty acid (PLFA) content.

**Results:**

For erythrocyte PLFA profiles, EPA, docosapentaenoic acid (DPA) and DHA percentage of total erythrocyte PLFA were significantly greater for the Ultimate Omega® group than for the MEG-3 group, at week 16 (*P* < 0.05), as were the EPA:arachidonic acid (AA) ratio, DHA:AA ratio and EPA+DHA:AA ratio. For serum PLFA profiles, increases in EPA:AA ratio and EPA+DHA:AA ratio were significantly greater at week 4 in the Ultimate Omega® group compared to the MEG-3 group (*P* < 0.05).

**Conclusions:**

These data suggest that the percentage of rTG in rTG fish oil preparations may evolve as a new chemoprofile/quality control marker that can influence its lipidomic pharmacodynamics. Additional investigations to assess the physiologic/vascular and metabolic/inflammasome responses to concentrated fish oil preparations differing in the percentage of rTG are warranted.

## Introduction

The use of supplements with high concentrations of omega-3 polyunsaturated fatty acids (N3PUFA) has become increasingly popular, delivering greater than 90% N3PUFA, most commonly as free fatty acids (FFA), ethyl esters (EE), or re-esterified triglycerides (rTG). The re-esterification process involves the transfer of N3PUFA from fish body oil (FBO) TG-bound fatty acid (FA) concentrates to ethanol, yielding EE concentrates which are then subjected to molecular distillation. The resulting distillate is enzymatically re-esterified into a TG form similar to natural FBO. The fidelity of re-esterification into a TG form is variable and may be a misnomer, as the glyceride profile of rTG supplements is approximately 55–60% TG, 38–42% diglycerides, and 1–3% monoglycerides [1]. Analysis of N3PUFA supplements has revealed mixtures of EE and glyceride forms in the same capsule [2], suggesting either less than complete re-esterification [3], or adulteration with EEs to augment the N3PUFA content, EEs being less costly/gram of N3PUFA than rTG forms.

Several intervention comparator studies of ≥ 12 weeks have indicated a relationship between lipid format and blood lipidomic response. Neubronner et al. randomized 150 statin therapy subjects with moderate hypertriglyceridemia into three groups: fish oil concentrate given as rTGs or EEs, delivering equal amounts of eicosapentaenoic acid (EPA) and docosahexaenoic acid (DHA), or a corn oil placebo [4]. The omega-3 index, which is a percentage of EPA+DHA (relative to total fatty acids) in erythrocyte (ERC) membranes [5], increased significantly in both groups treated with N3PUFA, but the increase was significantly greater in the rTG group than in the EE group. In contrast, West and colleagues [6] allocated 80 normolipidemic subjects to receive one of four different EPA+DHA supplements for twelve weeks: unmodified FBO (as native TG), rTG, FFA or EE, with each providing similar but not equal amounts of EPA+DHA (8–17% difference in EPA; 1–11% difference in DHA). No significant intergroup differences were seen in the concentration of EPA or DHA in plasma TG, non-esterified fatty acids, or phosphatidylcholine. ERC membrane concentrations of EPA and DHA were not measured.

Given the potential for incomplete re-esterification of acylglycerides into rTG forms of N3PUFA [2, 3], and the potential for EE forms to blunt the bioavailability and cellular membrane bio-incorporation of N3PUFA in certain subjects [4], it is of interest to determine if the degree of acylglyceride re-esterification has an influence on long term bio-incorporation. We hypothesized that the blood lipidomic response after sixteen weeks of supplementation with two different rTG LC3FA supplements, varying by degree of re-esterification, would significantly differ between treatments.

## Subjects and methods

### Study design

This study was a randomized, double-blind, parallel-design, single-center study conducted in accordance with local regulations, the International Conference on Harmonization E6 Guideline for Good Clinical Practice (GCP), and the Declaration of Helsinki. The study was sponsored by Nordic Naturals (Watsonville, CA) and conducted at Nutrasource Diagnostics (Guelph, Ontario). Ethical approval was obtained from an Institutional Review Board contracted by Nutrasource. The study was registered at ClinicalTrials.gov (Identifier #NCT02628483). Subjects were recruited in response to advertisements or from study site databases. Written, informed consent was obtained from all participants before any study-related activities. Recruitment began in March of 2016; the first subject was enrolled on March 15, 2016 and the last subject completed the study on December 8, 2016. The total intervention duration was 128 days, with two parallel groups: one group received a >96% TG form of N3PUFA (Ultimate Omega®; Nordic Naturals [UO]) and the other group received a <70% TG form of N3PUFA (MEG-3®, DSM Nutritionals, Heerlen, Netherlands [M3]).

### Fish oil products

UO bulk oil was manufactured and provided by Nordic Naturals; M3 bulk oil was manufactured by and purchased directly from DSM (Dartmouth, Nova Scotia, Canada) by a soft-gel dosage form manufacturer (Select Supplements, Carlsbad, California, USA). Percent TGs (%TGs) in the bulk oils were initially evaluated by each supplier. The soft-gel manufacturer measured %TGs in UO using The American Oil Chemists’ Society (AOCS) official method Ce 5b-89 [7] with high-performance liquid chromatography and mass spectrometry (HPLC-MS). DSM quantified %TGs in M3 using standard AOCS methods with HPLC. Each of the two bulk oils were encapsulated in identical soft gelatin capsules, with mixed tocopherols, rosemary extract, and natural lemon flavor added to promote shelf stability and create sensory-matched profiles. After encapsulation, the soft-gel manufacturer used AOCS official method Ce 5b-89 with HPLC-MS [7] to quantify %TGs in each of the finished/encapsulated products. Additional independent analyses measuring %TGs in both finished products were performed by a clinical research facility, using an in-house HPLC refractive index method (Diteba Laboratories Inc., Mississauga, Ontario, Canada). The UO capsules contained 376.4 mg EPA, 257.3 mg DHA, and 99.8 mg other omega-3 fatty acids, for a total of 733.5 mg total omega-3 fatty acids. The M3 capsules contained 304.5 mg EPA, 206.8 mg DHA and 83.2 mg other omega-3 fatty acids, for a total of 594.5 mg total omega-3 fatty acids (S1 and S2 Files).

### Subjects

The study enrolled healthy, non-smoking, normolipidemic adults, aged 18–35 years, with a body mass index (BMI) of 18.5 through 24.9 kg per m^2^. Subjects agreed to abstain from alcohol consumption for 24 hours prior to their clinic visits and to maintain stable body weight, level of physical activity, and dietary pattern. All blood samples were collected after an overnight fast. Female subjects were required to have had a negative urine pregnancy test and agree to use an effective method of birth control.

The following were the main exclusion criteria for this study: BMI ≥ 25 kg/m^2^; plasma TG of >400 mg/dL at screening; taking any prescription or non-prescription products that could affect any study endpoint, including blood lipid-lowering agents, any dietary supplements with N3PUFA, phytosterols, polyglucosamines, or lipid-binding agents in the previous 6 months; vegan diet, or consumption of an N3-rich diet (e.g. salmon, mackerel, herring) more than twice per month; unstable use (initiation or change in dose) of either anti-hypertensive or thyroid medications; use of weight-loss prescription medications, foods, or dietary supplements; pregnancy or lactation, or failure to agree to avoid pregnancy during the course of the study; history of blood-clotting disorders or use of anti-coagulation-inhibiting products (either prescription or non-prescription); presence or history of chronic diseases such as diabetes, cardiovascular, endocrine, gastrointestinal, renal, liver, neurological, cancer; uncontrolled hypertension (systolic, >140 mg Hg and /or diastolic >90 mg Hg); abnormal laboratory test result of clinical significance.

### Randomization and blinding

The subjects were randomly assigned 1:1 to either the UO or M3 group. The principal investigator, co-investigators, study personnel, and study participants were blinded to the treatment through the completion of the study. Blinding was maintained by labeling both products in a similar manner, differentiated only by their computer-generated randomization code, and with no other distinguishing information between the two products. In addition, the M3 capsules were similar in size and shape to the UO capsules. Allocation concealment was implemented by means of a restricted access drug dispensary unit within the CRO. An allocation schedule facilitated the release of subject supplements to trial personnel as scheduled.

### Study interventions

UO was provided as 1,000 mg soft-gel capsules (Batch 141179), each containing 325 mg EPA and 225 mg DHA, along with 90 mg of other FA, in a 90% re-esterified TG form. M3 was provided as 1,000 mg soft-gel capsules (Batch153143), each containing 300 mg EPA and 200 mg DHA, along with 90 mg of other FA in a 70% re-esterified TG form. Subjects in the respective groups were instructed to consume five capsules daily, taken orally with water at breakfast, providing a total daily amount of 2,750 mg EPA+DHA for UO and 2,500 mg EPA+DHA for M3.

The daily dosage level was selected in order to provide an easily comparable, statistically significant increase in ERC phospholipid (PL) N3PUFA content and serum PL N3PUFA content, using two similar chemical forms of EPA and DHA differing only in degree of re-esterification. The M3 oil was chosen because it approximated the EPA (92% w/w) and DHA (89% w/w) profile in UO. No dietary or lifestyle advice/counseling was given to study participants. Treatment compliance was evaluated at clinic visits by counting any unused capsules; minimum acceptable compliance was judged to be at least 80% of the intended dose. Study materials were provided in externally sealed, standard, high-density polyethylene bottles, each of which contained 150 capsules.

### Outcome measurements

The primary outcome measures were the intergroup comparisons of the ERC phospholipid fatty acid (PLFA) profiles at week 16 and the serum PLFA profiles at week 4. The secondary outcome measures were the comparisons of the ERC PLFA profiles at weeks 12 and 24, and blood lipids (i.e. TG, total cholesterol [TC], low-density lipoprotein cholesterol [LDL-C], and high-density lipoprotein cholesterol [HDL-C]) at week 24. Safety parameters, including vital signs, blood chemistry (Chem 20), biometrics and adverse events (AE), were also assessed at each visit. Intensity of AE was graded on a 3-point scale (mild, moderate, severe) and reported in detail in the study records. The Medical Dictionary for Regulatory Activities (MedDRA version 19.1) terminology was used to classify all AE with respect to System Organ Class and preferred term.

Serum samples and ERC samples were analyzed for PLFA profiles by GC-FID (Gas Chromatography-Flame Ionization Detector) using in-house developed and validated methods (Diteba Laboratories, Inc., Mississauga, Ontario, Canada). Venous blood samples were collected in serum separator tubes (SST) and EDTA (K2) tubes. Samples were processed according to standard clinical procedures for these collection tubes, and serum was collected from the SST tubes. ERC were collected from the EDTA tubes as a red cell pack after removal of the plasma and white blood cells. Samples were stored at -80°C prior to analysis. At time of analysis, frozen samples were thawed in an ice water bath. Serum samples were extracted with chloroform/methanol 2:1. Prior to injection, PLs were separated with a Hybrid SPE-PL cartridge (MilliporeSigma, St. Louis, MO). ERC samples were processed with BCl_3_-MeOH, heptane, methanol, with triheptadecanoin (C17:0) added to each ERC sample as an internal standard. Analyses for both serum PLFA and ERC PLFA were carried out using gas chromatography (Varian 3900, Palo Alto, CA) with a flameless ionization detector and a DB Wax, 30 m x 0.25 mm ID 0.15um column (Agilent Technologies, Santa Clara, CA). The resultant peak area for each FA identified was corrected to a percentage of total FA. Routine chemistry and hematology assays were performed by LifeLabs (Toronto, Ontario, Canada).

### Sample size estimation

Based on many previous omega-3 studies by the CRO, it was estimated that twenty-four (24) subjects per group would be required for acceptable statistical analysis. With an expected attrition rate of twenty-five (25) %, 30 subjects per group were required for enrollment. However, a formal sample size or power calculation was not performed.

### Statistical analyses

All calculations and statistical analyses were performed using SAS® (version 9.2 or later; SAS Institute, Cary, NC). Numerical efficacy endpoints were tested for significance by analysis of covariance (ANCOVA). The dependent variable was the post-baseline variable; the factor of interest was the UO group, and the value at baseline was the covariate. The baseline value was at visit 2, except for some laboratory tests (e.g. lipid profile, safety parameters) where baseline was at visit 1. The proportion of AE was compared between groups using Fisher’s Exact test or Chi-square test, as appropriate. All statistical tests were performed two-sided, and significance was accepted at the P = 0.05 level.

## Results

**Fig 1:**
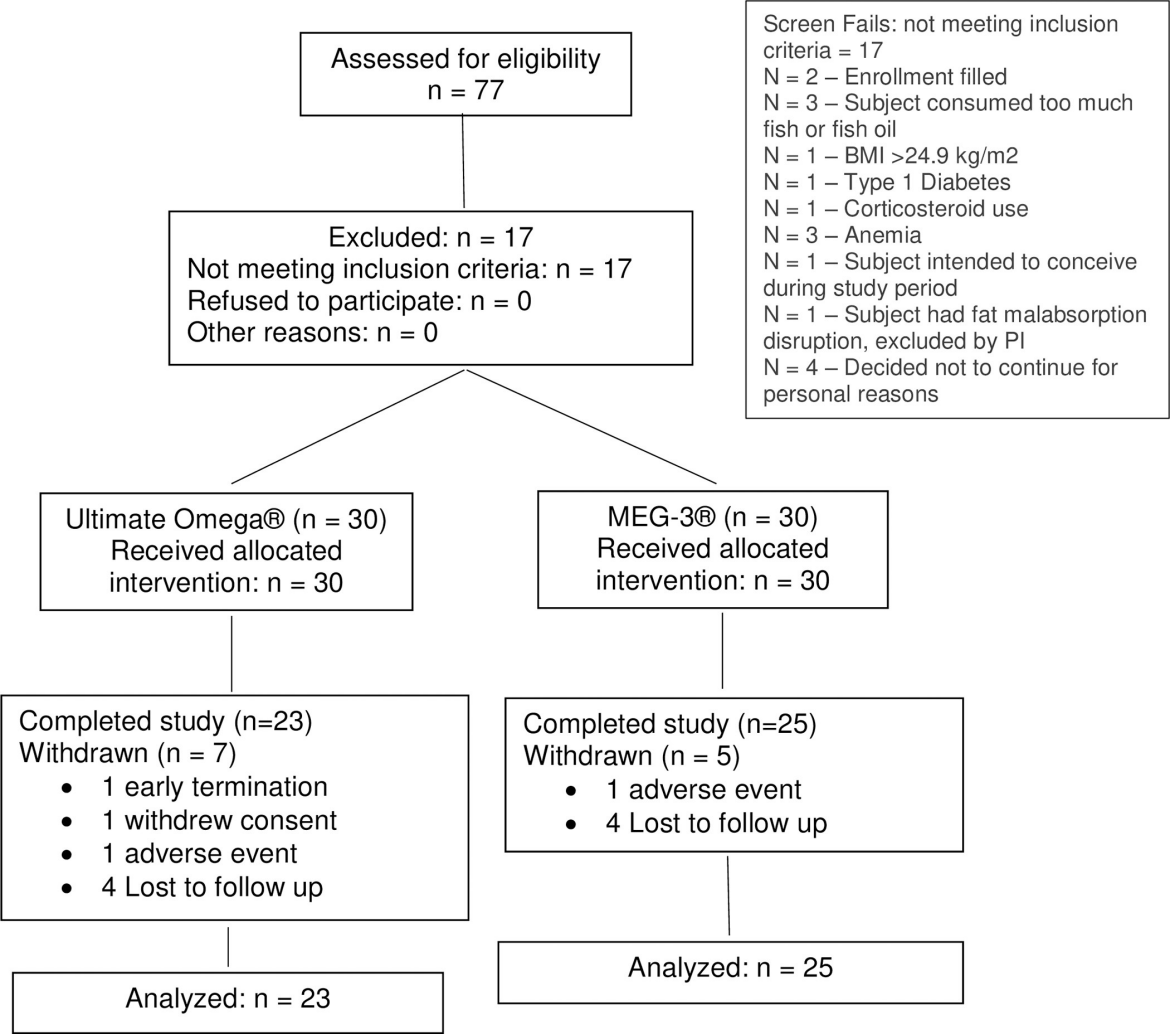
Consolidated Standards for Reporting of Trials (CONSORT) diagram.

The Consolidated Standards for Reporting of Trials (CONSORT) flow diagram shows the progress of the study subjects in Fig 1.

A total of sixty (60) individuals qualified for randomization (n = 30 for each intervention). During *post hoc* review of the unblinded dataset a number of subjects were deemed to have important protocol deviations with the potential to compromise efficacy assessment in relation to the outcome measures. Twelve subjects were excluded from the per protocol analysis set: seven subjects in the UO group were excluded due to protocol deviations: four subjects were lost to follow-up, one subject withdrew consent, one subject terminated participation prematurely, and one subject terminated due to an adverse event (urinary tract infection). Five M3 subjects were excluded due to protocol deviations: four were lost to follow-up and one subject terminated due to an AE unrelated to the study (left knee injury).

Table 1 describes the demographic data for the study subjects at baseline. The groups were equivalent in all demographic and anthropometric indices evaluated.

**Table 1:** Baseline demographic and other characteristics of enrolled subjects^1^.

Characteristic	Ultimate Omega (n = 30)	Meg-3® (n = 30)
Age, years	19–33 (avg = 23.9)	18–31 (avg = 24.2)
Sex [n (%)]		
Male	15 (50%)	15 (50%)
Female	15 (50%)	15 (50%)
Weight (kg)	69.5 ± 8.6	69.3 ± 9.2
Body Mass Index (kg/m^2^)^2^	22.8 ± 1.7	23.1 ± 1.6
Smoking		
Present (%)	0 (0%)	0 (0%)
Former (>6 months, %)	3 (10.0%)	0 (0%)
Absent (%)	27 (90.0%)	30 (100%)
Alcohol consumption		
Present (%)	26 (86.7%)	26 (86.7%)
Absent (%)	4 (13.3%)	4 (13.3%)
Systolic Blood Pressure (mm Hg)	115.5 ± 10.8	111.3 ± 12.1
Diastolic Blood Pressure (mm Hg)	71.5 ± 8.1	68.7 ± 11.1
Heart rate (beats per min)	71.5 ± 9.9	70.1 ± 11.9
Respiratory rate (breaths per min)	18.8 ± 2.2	19.5 ± 2.9
Oral temperature (° Celsius)	36.7 ± 0.3	36.6 ± 0.5

^1^Data are presented as the actual number (% of total number) or mean ± standard deviation, where appropriate.

^2^Body Mass Index: calculated as weight in kilograms divided by the square of height in meters.

Blood lipid indices were not influenced by either of the treatments in this young, normolipidemic population, as anticipated. Table 2 represents blood lipid results.

**Table 2:** Serum lipid results, by visit and group, with statistical comparisons.

Visit	Ultimate Omega	Meg-3®	
	[N] Mean ± Standard Deviation Median (Minimum, Maximum)		p-value^1^
	Triglycerides (TG) (mmol/L)		
Screening	[30] 0.95 ± 0.46 0.86 (0.32, 2.33)	[30] 0.83 ± 0.33 0.85 (0.38, 1.68)	
Week 24	[22] 0.97 ± 0.53 0.80 (0.53, 2.59)	[24] 0.88 ± 0.45 0.79 (0.37, 2.26)	0.738
	Total Cholesterol (TC) (mmol/L)		
Screening	[30] 4.20 ± 0.97 4.09 (2.68, 6.20)	[30] 4.37 ± 0.68 4.40 (3.02, 6.22)	
Week 24	[22] 4.26 ± 0.94 3.95 (3.07, 6.23)	[24] 4.49 ± 0.55 4.55 (2.98, 5.31)	0.639
	High Density Lipoprotein Cholesterol (HDL-C) (mmol/L)		
Screening	[30] 1.41 ± 0.40 1.39 (0.57, 2.51)	[30] 1.52 ± 0.41 1.44 (0.98, 2.55)	
Week 24	[22] 1.52 ± 0.34 1.51 (0.67, 2.35)	[24] 1.68 ± 0.42 1.65 (1.19, 2.66)	0.622
	TC: HDL-C Ratio		
Screening	[30] 3.00 ± 0.73 3.05 (0.90, 4.70)	[30] 3.05 ± 0.78 3.15 (1.60, 4.60)	
Week 24	[22] 2.87 ± 0.71 2.70 (1.90, 4.90)	[24] 2.81 ± 0.73 2.85 (1.40, 4.20)	0.477
	Low Density Lipoprotein Cholesterol (LDL-C) (mmol/L)		
Screening	[30] 2.36 ± 0.60 2.42 (1.52, 3.60)	[30] 2.49 ± 0.64 2.51 (0.97, 3.92)	
Week 24	[22] 2.29 ± 0.65 2.16 (1.23, 3.65)	[24] 2.41 ± 0.63 2.59 (0.69, 3.20)	0.945
	Non-HDL-C (mmol/L)		
Screening	[30] 2.79 ± 0.73 2.74 (1.72, 4.13)	[30] 2.86 ± 0.71 2.75 (1.16, 4.36)	
Week 24	[22] 2.73 ± 0.78 2.62 (1.47, 4.59)	[24] 2.80 ± 0.67 2.93 (0.91, 3.94)	0.991

^1^Between group statistical comparisons were made using the baseline (week 0) value as a covariate.

ERC PLFA profiles did not differ between groups at baseline (Table 3).

**Table 3:** Erythrocyte phospholipid fatty acid profiles at baseline and week 16^1^.

Lipid Parameter	Ultimate Omega (n, baseline = 30) (n, week 16 = 23)	Meg-3® (n, baseline = 30) (n, week 16 = 25)	p-value^2^
AA (% total fatty acids)			
Baseline	15.1 ± 2.1	15.7 ± 1.3	
Week 16	11.8 ± 1.0	12.6 ± 1.8	0.146
EPA (% total fatty acids)			
Baseline	0.57 ± 0.2	0.52 ± 0.2	
Week 16	3.11 ± 0.9	2.54 ± 0.8	0.046
DPA (% total fatty acids)			
Baseline	2.2 ± 0.5	2.2 ± 0.3	
Week 16	4.0 ± 0.6	3.6 ± 0.5	0.047
DHA (% total fatty acids)			
Baseline	3.76 ± 0.7	3.87 ± 0.8	
Week 16	6.30 ± 0.7	5.82 ± 0.8	0.040
EPA:AA ratio			
Baseline	0.04 ± 0.02	0.03 ± 0.01	
Week 16	0.27 ± 0.09	0.21 ± 0.08	0.049
DHA:AA ratio			
Baseline	0.25 ± 0.06	0.25 ± 0.06	0.048
Week 16	0.54 ± 0.09	0.48 ± 0.11	
EPA+DHA:AA ratio			
Baseline	0.29 ± 0.08	0.28 ± 0.06	0.039
Week 16	0.81 ± 0.17	0.69 ± 0.19	
AA:EPA ratio			
Baseline	29.7 ± 10.0	33.0 ± 10.1	0.243
Week 16	4.5 ± 3.2	6.8 ± 7.1	
AA:EPA+DHA ratio			
Baseline	3.6 ± 0.7	3.7 ± 0.8	0.054
Week 16	1.3 ± 0.3	1.6 ± 0.6	

AA: arachidonic acid; DHA: docosahexaenoic acid; DPA: docosapentaenoic acid; EPA: eicosapentaenoic acid.

^**1**^Data are means ± SD.

^*2*^Between group statistical comparisons were made using the baseline value as the covariate.

With respect to the primary outcome variables, the increases in the following parameters were significantly greater at week 16 in the UO group compared to the M3 group: EPA, docosapentaenoic acid (DPA), and DHA, each expressed as % of total ERC PLFA, as well as the EPA:arachidonic acid (AA) ratio, DHA:AA ratio and (EPA + DHA):AA ratio (*P* < 0.05; Table 3). Raw data for erythrocyte PLFA are available in S3 File.

Serum PLFA profiles were equivalent between groups at baseline (Table 4). There was a trend towards a significantly greater EPA level and DHA:AA ratio at week 4 for the UO group compared to the M3 group (p = 0.067 and p = 0.066, respectfully). The increases in EPA:AA ratio and (EPA + DHA):AA ratio were significantly greater at week 4 in the UO group compared to the M3 group (*P* < 0.05; Table 4). Raw data for serum PLFA are available in S4 File.

**Table 4:** Serum phospholipid fatty acid profiles at baseline and week 4^1^.

Lipid Parameter	Ultimate Omega (n, baseline = 30) (n, week 4 = 29)	Meg-3® (n, baseline = 30) (n, week 4 = 29)	p-value^2^
AA (% total fatty acids)			
Baseline	10.8 ± 2.4	11.1 ± 2.6	
Week 4	9.2 ± 1.7	9.8 ± 2.1	0.264
EPA (% total fatty acids)			
Baseline	0.75 ± 0.35	0.68 ± 0.27	
Week 4	4.2 ± 1.4	3.5 ± 1.3	0.067
DPA (% total fatty acids)			
Baseline	0.86 ± 0.22	0.83 ± 0.23	
Week 4	1.3 ± 0.3	1.2 ± 0.3	0.328
DHA (% total fatty acids)			
Baseline	2.9 ± 0.8	2.9 ± 0.6	
Week 4	5.2 ± 0.9	4.9 ± 1.1	0.161
EPA: AA ratio			
Baseline	0.07 ± 0.04	0.07 ± 0.03	
Week 4	0.48 ± 0.20	0.38 ± 0.16	0.040
DHA: AA ratio			
Baseline	0.28 ± 0.09	0.27 ± 0.07	
Week 4	0.59 ± 0.13	0.52 ± 0.15	0.066
EPA+DHA: AA ratio			
Baseline	0.35 ± 0.12	0.34 ± 0.09	
Week 4	1.07 ± 0.32	0.89 ± 0.29	0.036
AA:EPA ratio			
Baseline	16.5 ± 7.1	18.8 ± 8.9	
Week 4	3.0 ± 3.0	3.9 ± 5.1	0.521
AA:EPA+DHA ratio			
Baseline	3.1± 0.8	3.1 ± 0.7	
Week 4	1.1 ± 0.5	1.3 ± 0.7	0.177

AA: arachidonic acid; DHA: docosahexaenoic acid; DPA: docosapentaenoic acid; EPA: eicosapentaenoic acid.

^**1**^Data are means ± SD. ^**2**^Between group statistical comparisons were made using the baseline value as the covariate.

Both interventions were generally well tolerated, with no clinically significant changes appearing in either group. Neither blood chemistry results nor adverse events were clinically different between groups. No serious AE were reported. Table 5 lists AE that may have been related to the supplements.

**Table 5:** Adverse events suspected of being related to a product, by group.

Preferred terminology	Number of incidences of Adverse Events	
	Ultimate Omega	Meg-3®
Eructation	17	13
GERD	1	0
Dyspepsia	7	3
Abdominal Distension	4	1
Weight increase	3	3
Serum creatinine increase	1	0
Acne	0	2
Nausea	0	1
Abdominal pain	0	1
Abdominal discomfort	2	1
Seborrhoea	0	1
Breath odour	0	1

## Discussion

A variety of N3PUFA preparations are marketed internationally, varying in biomass source, e.g. fish liver or body oil, krill, or microalgal extracts and concentrates. Among FBO N3PUFA dosage forms displaying high percentages of EPA and DHA, the process of enrichment and concentration often employs transesterification and subsequent re-esterification into triglycerides (rTG). Re-esterification may not be a high fidelity process, resulting in FBO concentrates with variable percentages of rTGs and residual EEs [1–3].

Several long-term (≥ 12 weeks) studies have described varying bioavailability of EE forms of N3PUFA, relative to rTG forms [4, 6], with only one assessing ERC FA profiles [4]. One additional challenge for dosing with highly concentrated EE forms, e.g. Lovaza®, is the need for co-ingestion with fat-rich meals [8]. For certain individuals, TG forms of N3PUFA may be more effective than other forms with regard to chronic augmentation of lipidomic parameters. We believe the current investigation is the first long-term randomized controlled trial comparing EPA + DHA rTG preparations, differing with respect to percent rTGs and their lipidomic impact, specifically their bioincorporation into ERC membranes. Supplementation with a fish oil concentrate exhibiting a high % rTG composition profile (UO; >96% rTG) resulted in a greater increment in percent ERC fatty acid of EPA and DHA after sixteen weeks, compared to a product containing approximately 60% rTGs (M3).

These results may be partly explained by differences in the intestinal processing and absorption mechanics of TGs and EEs. Intraluminal digestion, micelle formation, transmembrane transport and enterocyte processing within the endoplasmic reticulum (ER) have been extensively studied and reviewed over the past 5 decades [9–13]. Current understanding reveals that TGs are quantitatively the most abundant component of daily human dietary fat, with the remainder comprising PL, cholesterol esters and FFA [14, 15]. When functioning normally, the gastrointestinal system digests and absorbs the majority of naturally structured TGs, with a fecal fat loss of approximately 5% or more indicative of malabsorption [16, 17]. After early catalytic activity by lingual and gastric lipase in the upper GI tract, most TGs containing long chain fatty acids (LCFA), such as EPA, DHA and other highly non-polar lipids, are emulsified into small volume particles by bile in the duodenum, under conditions influenced by FA chain length (hence polarity), degree of unsaturation, pH (thus ionization), temperature, partition coefficient and other factors [11]. In contrast, bile salts act to emulsify TGs with 1 remaining LCFA (a 2-monoglyceride [2-MG]), LC FFA and PL into various types of mixed micelles. These non-polar TGs and 2-MGs localized in particles and micelles are then disassembled into glycerol and polar-free LCFA by pancreatic lipase, colipase and cholesterol esterase acting within the unstirred water layer covering the surface of duodenal enterocytes [9, 11, 17–19]. Transport into the enterocyte follows, using both passive and transporter-mediated mechanisms [20].

Digestion and absorption of chemically modified EE LCFA appears to differ from natural dietary TGs or LCFA such as EPA and DHA. Removal of the synthetic ethyl group (CH_2_CH_3_) from the FA by pancreatic lipase has been shown to be 10 to 50 times slower than for natural fish oil TGs [21]. Furthermore, since animal and human studies demonstrate that EEs of EPA and DHA do not appear in the blood [22–24] after ethyl group cleavage and transport into the enterocyte, movement through the ER and/or subsequently into the blood or lymph may be limited by the availability of glycerol molecules required to resynthesize TGs. Indeed, absorption of EEs has been meaningfully increased when consumed with dietary fat that provided the glycerol molecules necessary for TG resynthesis [25]. Evidence thus suggests that compared to natural fish oil TGs, long-chain EEs experience less efficient digestion and movement through intestinal enterocytes.

The first critique of our study is the modest inequity of daily EPA and DHA dose from each test product, given that the UO group consumed 125 mg more per day of both EPA and DHA than the M3 group. The treatment selection was guided by the intention to compare two bulk oils with high but varying %rTG content. We elected to have the bulk oils encapsulated in the same facility and in an identical manner (antioxidants, flavor, soft-gel capsule size), and to dispense an identical daily capsule count so as to minimize unblinding of both subjects and investigators. Because the %N3PUFA content in M3 was slightly less than in UO, the daily amount of N3PUFA ingested differed marginally between groups. Although this marginal difference would not be expected to drastically change the results, future studies should attempt to more precisely match EPA and DHA levels.

Another critique of our study is the absence of dietary n-3 FA intake data collected *during* the study. During the screening process, prospective subjects were asked to complete a food frequency questionnaire. Although we found no between-group differences in n-3 intakes, nor in EPA + DHA intakes before subjects were randomized to treatment, we cannot rule out the possibility that dietary fat intake may have changed during the intervention period. Targeted reductions in dietary n-6 FA intakes (linoleic acid and AA) over twelve weeks, without a significant increase in EPA or DHA intakes, can elicit significant increases in ERC %EPA and %DHA content among free-living women and men [26]. However, because a restricted N-6 diet requires assiduous adherence to dietary modifications e.g. limited consumption of egg yolks, meat, most poultry and certain seafood items, it is unlikely that our trial participants pursued such adjustments, given a lack of specific instructions in that regard.

## Conclusion

We observed greater increases in EPA- and DHA-containing lipidomic species after 1 and 4 months of supplementation from a higher % rTG FO preparation (UO) containing a very similar amount of EPA and DHA, in young, normolipidemic subjects, in comparison to a lower %rTG FO preparation (Meg-3®). These data suggest that, like EE vs. rTG compositions, % rTGs in rTG FO preparations may evolve as a new chemoprofile/quality control marker that can influence its lipidomic pharmacodynamics. Given the widespread attention to fish oil supplements, and the fact that the global market value for fish oil is estimated to exceed US$23 billion [27], additional investigations in dyslipidemic and statin-treated populations appear warranted. Moreover, investigations to assess the physiologic/vascular and metabolic/inflammasome responses to concentrated FO preparations differing in % rTGs are encouraged.

## Supporting information

S1 Checklist CONSORT 2010 checklist of information to include when reporting a randomised trial*. (DOC)

S1 File Certificate of analysis for Ultimate Omega®. (PDF)

S2 File Certificate of analysis for Meg-3®. (PDF)

S3 File Individual red blood cell fatty acid raw data. (XLSX)

S4 File Individual serum fatty acid raw data. (XLSX)

S5 File Protocol. (PDF)
