# Supplementary material for: Comparative membrane incorporation of omega-3 fish oil triglyceride preparations differing by degree of re-esterification: A sixteen-week randomized intervention trial
Source: PLoS One. 2023 Jan 27;18(1):e0265462. doi: 10.1371/journal.pone.0265462 (PMC9882700; doi:10.1371/journal.pone.0265462)
Supplement: S1 File — (PDF) [file pone.0265462.s002.pdf]

NORDIC NATURALS

Certificate of Analysis

Product: Ultimate Omega 60ct.	Bulk Lot Nr.: 140851	Bottle Lot Nr.: 141179
Manufacture Date: June, 2014	Item Nr.: CAN-71790	Shelf Life: Three years from manufacture date

Oxidation	Test Method	Limits	Assay Result
ACID	AOCS Cd 3d-63	NMT 3.0 KOH/g	0.2 KOH/g
PEROXIDE	AOCS Cd 8b-90	NMT 5.0 meq/kg	3.2 meq/kg
ANISIDINE	AOCS Cd 18-90	NMT 20.0 meg/kg	4 meq/kg
TOTOX (TOTAL OXIDATION)	Calculation	NMT 26.0 meq/kg	10.4 meq/kg

Heavy Metals	Test Method	Limits	Assay Result
ARSENIC	USEPA 305.1, 200.7, 200.8	NMT 0.1 mg/kg (ppm)	< 0.05 ppm
CADMIUM	USEPA 305.1, 200.7, 200.8	NMT 0.1 mg/kg (ppm)	< 0.01 ppm
LEAD	USEPA 305.1, 200.7, 200.8	NMT 0.1 mg/kg (ppm)	< 0.02 ppm
MERCURY	USEPA 245.6	NMT 0.1 mg/kg (ppm)	< 0.005 ppm

Environmental Toxins	Test Method	Limits	Assay Result
POLYCHLORINATED BIPHENYLS (PCBs)	USEPA 1668 A/C	NMT 0.09 mcg/kg (ppm)	0.003 ppm
DIOXIN-LIKE PCBs (non-ortho & mono-ortho)	USEPA 1668 A/C	NMT 1.0 pcg/g (ppt)	0.01 ppt
DIOXINS & FURANS (WHO TEQ)	USEPA 1613B	NMT 2.0 pcg/g (ppt)	0.2 ppt

Microbial Analysis	Test Method	Limits	Assay Result
PLATE COUNT	USP <2021>	<1000 cfu/g	Negative
STAPHYLOCOCCUS AUREUS	USP <2022>	Absent	Absent
ESCHERICHIA COLI	USP <2022>	Absent	Absent
SALMONELLA	USP <2022>	Absent	Absent
YEAST & MOLD	USP <2021>	<100 cfu/g	Negative

Fatty Acid Profile	Test Method	Label Claim (mg/capsule)	Assay Result
EICOSAPENTAENOIC ACID (EPA ; 20:5 (n-3))	AOCS CE 1b-89	325 mg	376.4 mg
DOCOSAHEXAENOIC ACID (DHA ; 22:6 (n-3))	AOCS CE 1b-89	225 mg	257.3 mg
TOTAL OMEGA-3	AOCS CE 1b-89	640 mg	733.5 mg
OTHER OMEGAS	AOCS CE 1b-89	90 mg	99.8 mg

Radioactivity	Test Method		Assay Result
Radioactivity - Cesium 134	JCRCI: Radioactivity		<3 Bq/Kg
Radioactivity - Cesium 137	JCRCI: Radioactivity		<3 Bq/Kg
Radioactivity - Iodine 131	JCRCI: Radioactivity		<3 Bq/Kg

Prepared By	Reviewed By
[signature] Quality Assurance	Gilda Pedigo Quality Assurance

111 Jennings Drive, Watsonville, CA 95076
Phone 831.724.6200 - Fax 831.724.6600 - Toll Free 800.662.2544 - admin@nordicnaturals.com - nordicnaturals.com
