# Supplementary material for: Comparative membrane incorporation of omega-3 fish oil triglyceride preparations differing by degree of re-esterification: A sixteen-week randomized intervention trial
Source: PLoS One. 2023 Jan 27;18(1):e0265462. doi: 10.1371/journal.pone.0265462 (PMC9882700; doi:10.1371/journal.pone.0265462)
Supplement: S2 File — (PDF) [file pone.0265462.s003.pdf]

Select Supplements
INCORPORATED

Bottled Product Certificate of Analysis

Item: NA	Product: MEG-3, 150 ct. bottle		Page: Page 1 of 1
Version / index: 09.10.15 / 01	Supersedes: **.**.**/**		UPC Bar Code #: NA
Nordic Sku #: NA			Lot Number: 153521<br>Expiration date: 07.18
Bulk softgel lot # used: 153143	Label Rev.: NA		PO #: NA

Quality Data:

Item	Specification	Method	Result
Bottle	300 cc HDPE white, Item # 15012	Batch record	Conforms
Capsule	ProOmega softgel, Item # 31262-NNM	Batch record	Conforms
Bottle count	150 capsules	Physical Count	Conforms
Cap	45-400 Lift-n-Peel, ribbed, white, w/ foil induction seal, Item # 15048	Visual/Batch record	Conforms
Neck Band	77 mm LFW, seamless, clear, no print, w/ vertical perforation, Item # 54079	Batch record	Conforms
12-pack shipper	For bottle Item # 15012, Item # 54084	Batch record	Conforms
Divider	For 12-pack shipper for bottle Item # 15012, Item #54085	Batch record	Conforms
Total Aerobic Count	n.m.t. 1000 cfu/g	TM-7141	5 cfu/g
Yeast & Mold	n.m.t. 100 cfu/g	TM-7144	<10 cfu/g
Salmonella	Negative	TM-7143	Negative
E. Coli	Negative	TM-7140	Negative
Staph. aureus	Negative	TM-7142	Negative
Total coliforms	Negative	TM-7140	Negative

Disposition:

☑ Approved ☐ Rejected

By: hauheng (Quality Assurance) Date: 09.10.15

5800 Newton Drive • Carlsbad, CA 92008 • Phone: (760) 431-7509 • Fax: (760) 804-8071
www.selectsupplements.com

NSF GMP for Sport™ www.nsf.org
NSF GMP Registered www.nsf.org

Select Supplements INCORPORATED

Product Certificate of Analysis

Report Date: **08.31.15**
Report Number: **150243**

Product Identity:

**Material Number:**	NA		
**Material Name:**	MEG-3 / (FORCE-study) softgel NN	Best-by date:	07.2018
**Batch Number:**	153143		
**Production date:**	07.2015	Cust. PO # :	
**Description:**	Yellow liquid in a clear softgel, with lemon aroma, size 20 oblong		

Quality Attribute	Specification	Method	Results
Fill color	Yellow	Visual	Conforms
Shell color	Clear, with lemon aroma	Visual	Conforms
Size	20 oblong	Visual	Conforms
Average shell weight	Target 340 - 440 mg/cap	TM-1030/1032	407.4 mg/cap
Average fill weight	n.l.t. 1032.5 mg/cap	TM-1030/1032	1044.0 mg/cap
Weight variation	Meets USP requirements	TM-1030	Conforms
Disintegration	n.m.t. 30 minutes	TM-3410	Conforms
Peroxide value	Report Only	TM-6030	1.7 meq/kg
Total aerobic plate count	< 1,000 cfu/g	TM-7141	10 cfu/g
Yeast & Mold	< 100 cfu/g	TM-7144	<10 cfu/g
Total Coliforms	Negative	TM-7140	Negative
Salmonella	Negative	TM-7143	Negative
E. coli	Negative	TM-7140	Negative
Staphylococcus aureus	Negative	TM-7142	Negative
Active ingredient			
**MEG-3 Oil**			
EPA	n.l.t. 300 mg/cap	TM-9010	304.5 mg/cap
DHA	n.l.t. 200 mg/cap	TM-9010	206.8 mg/cap
Total Omega-3	n.l.t. 540 mg/cap	TM-9010	594.5 mg/cap
Other Omega-3	n.l.t. 40 mg/cap	TM-9010	83.2 mg/cap

Other Ingredients: Gelatin, Glycerin, Lemon flavor, Water, Rosemary extract, Vitamin E.

Select Supplements, Inc.

[signature]

(Quality Assurance)

08.31.15

(Date)

NSF GMP for Sport™ www.nsf.org

5800 Newton Drive • Carlsbad, CA 92008 • Phone: (760) 431-7509 • Fax: (760) 804-8071
www.selectsupplements.com

NSF GMP Registered www.nsf.org

Select Supplements
INCORPORATED

Bottled Product Certificate of Analysis

Item: NA	Product: MEG-3, 150 ct. bottle		Page: Page 1 of 1
Version / index: 09.10.15 / 01	Supersedes: **.**.**/**		UPC Bar Code #: NA
Nordic Sku #: NA			Lot Number: 153521<br>Expiration date: 07.18
Bulk softgel lot # used: 153143	Label Rev.: NA		PO #: NA

Quality Data:

Item	Specification	Method	Result
Bottle	300 cc HDPE white, Item # 15012	Batch record	Conforms
Capsule	ProOmega softgel, Item # 31262-NNM	Batch record	Conforms
Bottle count	150 capsules	Physical Count	Conforms
Cap	45-400 Lift-n-Peel, ribbed, white, w/ foil induction seal, Item # 15048	Visual/Batch record	Conforms
Neck Band	77 mm LFW, seamless, clear, no print, w/ vertical perforation, Item # 54079	Batch record	Conforms
12-pack shipper	For bottle Item # 15012, Item # 54084	Batch record	Conforms
Divider	For 12-pack shipper for bottle Item # 15012, Item #54085	Batch record	Conforms
Total Aerobic Count	n.m.t. 1000 cfu/g	TM-7141	5 cfu/g
Yeast & Mold	n.m.t. 100 cfu/g	TM-7144	<10 cfu/g
Salmonella	Negative	TM-7143	Negative
E. Coli	Negative	TM-7140	Negative
Staph. aureus	Negative	TM-7142	Negative
Total coliforms	Negative	TM-7140	Negative

Disposition:

☑ Approved ☐ Rejected

By: [signature] (Quality Assurance) Date: 09.10.15

NSF GMP for Sport™ www.nsf.org

5800 Newton Drive • Carlsbad, CA 92008 • Phone: (760) 431-7509 • Fax: (760) 804-8071
www.selectsupplements.com

NSF GMP Registered www.nsf.org
