# Supplementary material for: Comparative membrane incorporation of omega-3 fish oil triglyceride preparations differing by degree of re-esterification: A sixteen-week randomized intervention trial
Source: PLoS One. 2023 Jan 27;18(1):e0265462. doi: 10.1371/journal.pone.0265462 (PMC9882700; doi:10.1371/journal.pone.0265462)
Supplement: S5 File — (PDF) [file pone.0265462.s006.pdf]

CLINICAL PROTOCOL

A randomized, double-blind, parallel clinical trial to compare the relative efficacy of two triglyceride forms of fish oil, Ultimate Omega®, and a comparator product, in improving red blood cell fatty acid profiles in healthy adults (FORCE Study)

Sponsor: Nordic Naturals
111 Jennings Drive
Watsonville, CA 95076
Phone: (831) 724-6200

Sponsor Representative: Scott Minton, MS, PhD
Scientific Advisor
Nordic Naturals
111 Jennings DriveF
Watsonville, CA 95707
Phone: (949) 310-3455
Email: sminton@nordicnaturals.com

CRO: Nutrasource Diagnostics Inc.
120 Research Lane, Suite 203
Guelph, ON N1G0B4
Phone: (519) 341-3360

Principal Investigator: Anthony Bier, MD
Nutrasource Diagnostics Inc.
120 Research Lane, Suite 203
Guelph, ON N1G0B4
Phone: (519) 341-3360

Sub-Investigator: Carolina Bonilla, MD, PhD
Nutrasource Diagnostics Inc.
120 Research Lane, Suite 203
Guelph, ON N1G0B4
Phone: (519) 341-3360

SPONSOR'S APPROVAL / SIGNATURE

I have read and understand the attached protocol entitled "**A randomized, double-blind, parallel clinical trial to compare the relative efficacy of two triglyceride forms of fish oil, Ultimate Omega®, and a comparator product, in improving red blood cell fatty acid profiles in healthy adults (FORCE Study)"** dated Jan 22, 2016 and agree to abide by all described protocol procedures.

As the sponsor, Nordic Naturals transfers some of the sponsor's responsibility to Nutrasource Diagnostics Inc., as documented in the Transfer of Regulatory Obligations document dated 18 Jul 2014, which ensures that this protocol is conducted as per all applicable regulatory and international guidelines for conducting clinical trials.

I agree that I have reviewed the ICH Guideline for Good Clinical Practice and am aware of my obligations and responsibilities as a sponsor.

Authorized Sponsor Representative: ______________________________

Signature: ______________________________

Date: ______________________________

INVESTIGATOR'S AGREEMENT/SIGNATURE

I have read and understand the attached protocol entitled **"A randomized, double-blind, parallel clinical trial to compare the relative efficacy of two triglyceride forms of fish oil, Ultimate Omega®, and a comparator product, in improving red blood cell fatty acid profiles in healthy adults (FORCE Study)"** dated Jan 22, 2016 and agree to abide by all described protocol procedures.

I agree that it contains all the necessary details for me and my staff to carry out the study, which I agree to conduct and supervise as described therein.

I will ensure that all individuals responsible to me who assist in the study are provided with the protocol and are fully informed regarding the procedures needed.

I agree to follow ICH Guideline for Good Clinical Practice as it pertains to Investigators. I agree to ensure that the regulatory requirements for obtaining written informed consent from study participants are met. I agree to maintain adequate and accurate study records, and to make these records available for inspection in accordance with applicable regulatory requirements.

I understand that if any questions arise during the course of the study, I will contact Nordic Naturals.

Site Principal Investigator Signature: ______________________________

Print Site Principal Investigator Name: ______________________________

Date: ______________________________

PROTOCOL SYNOPSIS

**Protocol Title:**	**A randomized, double-blind, parallel clinical trial to compare the relative efficacy of two triglyceride forms of fish oil, Ultimate Omega®, and a comparator product, in improving red blood cell fatty acid profiles in healthy adults (FORCE Study)**
**Protocol Number:**	1007-008-PRO-040414
**Sponsor:**	Nordic Naturals
**Phase of Development:**	Phase II
**Methodology:**	Randomized, Double-blind, Parallel
**Study Duration:**	The expected duration for each individual participant who is enrolled and subsequently randomized to intervention is estimated to be six months.
**Study Centre(s):**	Nutrasource Diagnostics Inc.,<br>120 Research Lane, Suite 203,<br>Guelph, ON
**Objectives:**	The primary objective of this clinical trial is to assess the relative efficacy of a 90% rTG to another TG form of fish oil
**Sample Size:**	60 healthy adults (30 male and 30 female)
**Investigational Product (IP) Dose, Route, Regimen**	**Brand Name(s):** Ultimate Omega®<br>**NPN:** 80003338<br>**Common Name:** Fish Oil<br>**Recommended Route of Administration:**<br>Oral<br><br>**Recommended Dose:**<br>5 capsules<br><br>**Medicinal Ingredients (per capsule):**<br>Fish Oil 1000mg<br>(325mg EPA, 225mg DHA and 90mg Other Omega-3 Fatty Acids)<br><br>**Dosing Instructions:**<br>Subjects will be instructed to take 5 capsules in the morning with food daily for 6 months, starting the day following the randomization visit.
**Comparator Product Dose, Route, Regimen**	**Brand Name(s):** Meg-3™<br>**Common Name:** Fish Oil<br>**Recommended Route of Administration:**<br>Oral<br><br>**Recommended Dose:**<br>5 capsules<br><br>**Medicinal Ingredients (per capsule):**<br>Fish Oil 1000mg<br>(300mg EPA and 200mg DHA)<br><br>**Dosing Instructions:**

	Subjects will be instructed to take 5 capsules in the morning with food daily for 6 months, starting the day of the randomization visit.
**Overview of Study Design**	This will be a randomized, double-blind, parallel study design. Healthy adults from the city of Guelph, Ontario (and surrounding communities), will be recruited to take part in this study. Individuals who satisfy the inclusion/exclusion requirements will be randomly assigned to one of two treatments.
**Inclusion Criteria:**	I1. Male or female adults aged 18 – 35 (inclusive)<br>I2. Healthy as determined from medical history<br>I3. Non-smoker, or ex-smoker ≥6 months<br>I4. Body mass index 18.5 – 24.9 kg/m^2 (inclusive)<br>I5. Female participants of childbearing potential (i.e. not surgically sterilized or post-menopausal greater than one year) must have negative urine pregnancy test and must be using an effective birth control method , defined as:<br>• Continuous use of oral or long acting injected contraceptive for at least 2 months prior to study entry , or<br>• Use of an intra-uterine device or implantable contraceptive, or<br>• Use of double barrier methods of birth control, or<br>• Abstinence from heterosexual intercourse<br>I6. Willing to avoid alcohol consumption for 24 h prior to every clinic visit<br>I7. Willing to maintain a stable body weight, activity level and dietary pattern except for use of the study products, as directed<br>I8. Willing and able to provide informed written consent
**Exclusion Criteria:**	E1. Individuals taking prescription or non-prescription health products that may affect the study endpoint (e.g. corticosteroids, prescription anti-inflammatory drugs, blood lipid-lowering drugs (e.g. statins, fibrates, bile acid exchanger resin, phytosterols, niacin or its analogues, carnitine, etc.) in the previous 6 months<br>E2. Individuals taking any supplements with n-3 FAs, phytosterols, polyglucosamines (Chitosan) or other lipid-binding ingredients in the previous 6 months<br>E3. Individuals who consume omega-3 fatty acid rich fish (salmon, mackerel, herring) more than twice per month<br>E4. Unstable use (i.e.initiation or change in dose) of antihypertensive medications or thyroid hormone replacement medications within 3 months prior to visit 1<br>E5. Use of any weight-loss programs or weight-loss medications (prescription or over-the counter) including, but not limited to,

	lipase inhibitors, within 6 months prior to visit 1 and throughout the study<br>E6. Pregnancy or lactation, or participant unwilling to take appropriate contraceptives for the duration of the study<br>E7. History of blood clotting disorders or use of coagulation-inhibiting drugs (e.g. warfarin)<br>E8. Presence of major diseases such as diabetes, gastrointestinal, endocrine, cardiovascular, renal, or liver disease<br>E9. History of neurological disease (e.g. Parkinson's disease, stroke, traumatic brain injury, etc.)<br>E10. History of cancer (excluding non-melanoma skin cancer and basal cell carcinoma) in the past 5 years<br>E11. Uncontrolled hypertension defined as a seated resting systolic blood pressure ≥140 mmHg and/or diastolic blood pressure ≥90 mmHg<br>E12. Abnormal laboratory test results of clinical significance, including, but not limited to ALT or AST ≥1.5X the upper limit of normal at screening (visit 1)<br>E13. TG ≥400 mg/dL (≥4.52mmol/L) at screening (visit 1)<br>E14. Presence or history (past 6 months) of alcohol or drug abuse; alcohol use of >2 standard alcoholic drinks per day<br>E15. Participant has a known allergy or intolerance to fish/fish oil or any of the ingredients in the test products<br>E16. Participant is vegan<br>E17. Participant is unwilling or unable to abide by the requirements of the protocol<br>E18. Any condition that would interfere with the participant's ability to comply with study instructions, might confound the interpretation of the study, or put the participant at risk<br>E19. Participant has taken an investigational medicine or has participated in a research study within 30 days prior to first study visit
**Criteria for Efficacy Evaluation:**	Red blood cell fatty acid profile;<br>Serum phospholipid fatty acids;<br>Total cholesterol, HDL-C, LDL-C and triglycerides
**Criteria for Safety Evaluation:**	Adverse Events (AEs)
**Planned Interim Analysis:**	When approximately 100% of participants have completed Visit 3 (Day 28), an interim analysis to determine changes in serum phospholipid fatty acids will be performed by research staff not involved in the conduct of the clinical trial.

**Statistical Methods:**	Numerical efficacy endpoints will be formally tested for significance by analysis of covariance. The dependent variable will be the post-baseline value; the factor of interest will be the treatment group (product) and value at baseline (usually visit 2, except for some laboratory tests [e.g. lipid profile and safety parameters] where baseline is visit 1) will be the covariate.<br><br>The proportion of adverse events will be compared between groups using Fisher's Exact test or Chi-square test, as appropriate.<br><br>All calculations and analyses will be performed using SAS version 9.2 or higher. For all statistical inferences, the tests will be performed at the two-sided, 0.05 level of significance.

Table of Contents

Sponsor's Approval / Signature 2
Investigator's Agreement/Signature 3
Protocol Synopsis 4
List of Tables 11
List of Figures 11
List of Abbreviations 12
1 INTRODUCTION 14
1.1 Background 14
1.2 Investigational Product 15
1.3 Risk/Benefits 16
1.3.1 Potential Risks 16
1.3.2 Known Potential Benefits 16
2 TRIAL OBJECTIVES AND PURPOSE 16
2.1 Primary Objectives 16
2.1 Secondary Objectives 16
2.2 Additional Objectives 16
3 TRIAL DESIGN 17
3.1 Overall Study Design 17
3.1.1 Study Centres 17
3.1.2 Study Duration 17
3.2 Study Flow Chart 17
3.3 Schedule of Events 18
3.4 Narrative of Study Procedures 19
3.4.1 Visit 1 (-2 to -30 days prior to Day 0 – Screening Visit) 19
3.1.1 Visit 2 (Day 0) [Baseline; Randomization] 20
3.1.2 Visit 3 (Day 28±2) 20
3.1.3 Visit 4 (Day 84±2) 21
3.1.4 Visit 5 (Day 112±2) 21
3.1.5 Visit 6 (Day 168±2) 22
3.1.6 Unscheduled Visits 22
3.1.7 Early Termination/Discontinuation Visit 22
3.2 Diagnostic Tests 23
3.3 Allocation to Treatment 23
3.4 Stopping Rules/Discontinuation 23

3.5 Concomitant Medication(s) 24
3.5.1 Permitted Medication(s) 24
3.5.2 Prohibited Medication(s) 24
3.5.3 Washout Period 24
3.6 Measures to Minimize Bias 24
4 SELECTION AND WITHDRAWAL OF SUBJECTS 25
4.1 Number of Study Volunteers 25
4.2 Inclusion Criteria 25
4.3 Exclusion Criteria 26
4.4 Withdrawal Criteria 27
5 INVESTIGATIONAL PRODUCT 28
5.1 Treatment Groups 28
5.2 Description of IP 28
5.1 Description of Comparator Product 28
5.2 Investigational Product Packaging and Labeling 29
5.3 Dosage and Administration of IP 29
5.4 Storage 29
5.5 IP Accountability 29
5.6 Blinding 29
5.7 Unblinding 30
6 ASSESSMENT OF EFFICACY 30
6.1 Primary Endpoints 30
6.2 Secondary Endpoints 30
7 ASSESSMENT OF SAFETY 30
7.1 Adverse Events (AEs) 30
7.1.1 AE Reporting 31
7.1.2 Unexpected Adverse Reaction 31
7.1.3 Plan for Grading Adverse Events: 31
7.1.4 Plan for Attribution of Adverse Events: 31
7.1.5 Plan for Determining Seriousness of Adverse Events: 32
7.1.6 Serious Adverse Event (SAE) Reporting 33
7.1.7 Follow-up of AEs 34
7.2 Vital Signs 34
7.3 Biometrics 34
7.4 Hematology and Clinical Chemistry 35

8 STATISTICS 35
8.1 Planned Interim Analysis 35
8.2 Sample Size 35
8.3 Analytical Populations 35
8.3.1 Intent-To-Treat Population 35
8.3.2 Safety Population 35
8.4 Statistical Methods 35
8.5 Safety Analysis 36
8.6 Handling of Missing or Spurious Data 37
8.7 Premature Discontinuation Description 37
8.1 Protocol Deviations and Violations 37
8.2 Termination of the Trial 37
9 QUALITY CONTROL AND QUALITY ASSURANCE 37
9.1 Auditing 37
9.2 Monitoring 37
10 ETHICAL CONSIDERATIONS 38
10.1 Ethical Principles 38
10.1.1 Informed Consent 38
10.1.2 Research Ethics Board (REB) 39
10.2 Incentives and Benefits for the Subjects 39
11 DATA HANDLING AND RECORD KEEPING 39
12 PROTOCOL AMENDMENTS AND END OF STUDY REPORT 40
12.1 Protocol Amendments 40
12.2 End of Study Report(s) 40
13 DATA COLLECTION METHODS 41
14 FINANCING AND INSURANCE 41
15 PUBLIC DISCLOSURE AND PUBLICATION POLICY 41
16 REFERENCES 42

LIST OF TABLES

Table 1 Schedule of events by visit, including all procedures 18

LIST OF FIGURES

Figure 1 Study Flow Chart........ 17

LIST OF ABBREVIATIONS

Abbreviation/Term	Definition
AA	Arachidonic Acid
AE	Adverse Event
ALT	Alanine transaminase
ANCOVA	Analysis Of Covariance
AST	Aspartate aminotransferase
BMI	Body Mass Index
BUN	Blood urea nitrogen
CBC	Complete blood count
CIOMS	Council for International Organizations of Medical Sciences
Cl	Chloride
CRF	Case Report Form
CRO	Contract Research Organization
CVD	Cardiovascular Disease
DHA	Docosahexaenoic Acid
dL	Decilitres
DPA	Docosapentaenoic Acid
eGFR	Estimated Glomerular Filtration Rate
EPA	Eicosapentaenoic Acid
FA	Fatty Acid
FDA	Food and Drug Administration
GCP	Good Clinical Practice
G	Grams
h	Hour
HDL-C	High Density Lipoprotein Cholesterol
ICF	Informed Consent Form
ICH	International Conference on Harmonisation
i.e.	That is
IP	Investigational Product
IRB	Institutional Review Board
K	Potassium
Kg	Killogram
LCPUFA	Long Chain Polyunsaturated Fatty Acid
LDL-C	Low Density Lipoprotein Cholesterol
M	Metre
mg	Milligrams
mL	Millilitres
mmHg	Millimetres Mercury
Na	Sodium
NDI	Nutrasource Diagnostics Incorporated
NHP	Natural Health Product
NNHPD	Non-Prescription and Natural Health Products Directorate
ω-3 FA	Omega-3 fatty acids
OTC	Over-the-counter

Abbreviation/Term	Definition
PI	Principal Investigator
RBC	Red Blood Cell
REB	Research Ethics Board (synonymous with IRB)
SAE	Serious Adverse Event
SF-12	Short Form-12
TG	Triglyceride
TMF	Trial Master File
TPD	Therapeutics Products Directorate
vWF	vonWillebrand Factor

1 INTRODUCTION

The contents of this document describe the clinical trial procedures that will be followed by the research team and includes information on the access to source data/documents, ethics, data handling and record keeping, as well as procedures for the response to adverse events and/or stopping rules.

1.1 Background

The health benefits of omega-3 fatty acids (ω-3 FAs) have been extensively examined in many published studies, and these benefits have been observed in patients with a diversity of conditions and diseases, including cardiovascular disease (e.g. atrial fibrillation, atherosclerosis, thrombosis, inflammation, sudden cardiac death, etc.), age-related cognitive decline, periodontal disease, rheumatoid arthritis, etc. (1-9).

These fatty acids may also be beneficial to healthy individuals, in terms of preventative health benefits. In a 2014 published statement, the American Heart Association notes that "*Omega-3 fatty acids benefit the heart of healthy people…*" (10). One of the established biomarkers of omega-3 status, the whole blood OmegaSpot™ is a combined measure of blood eicosapentaenoic acid (EPA) + docosapentaenoic acid (DPA) + docosahexaenoic acid (DHA) levels, and quartiles of this biomarker are inversely linked to risk of sudden death (11). From this biomarker, three other biomarker scores have been derived. The Omega-3 Serum Equivalence Score is inversely associated with heart disease risk reduction (12), the EPA+DHA Serum Equivalence Score is inversely correlated with risk of death from fatal ischemic heart disease (13), and the Omega-3 Red Blood Cell Equivalence Score, also known as the Omega-3 Index, is inversely linked to protection against sudden myocardial infarction (14).

Shahar et al. (1993), reported that the dietary intake of ω-3 FAs was inversely associated with blood levels of several coagulation factors, and that one serving per day of fatty fish reduced the levels of fibrinogen, factor VIII, and von Willebrand factor (vWF) by 2.9 mg/dL, 3.3% and 2.7%, respectively (15). The hemostatic profile is an important predictor of cardiovascular disease (CVD); reducing the levels of hemostatic biomarkers such as fibrinogen, Factor VIII and factor vWF may produce a hypocoagulable profile and thus reduce CVD risk in normal individuals.

Sudden cardiac death may be idiopathic in some individuals, but in others it has been linked to electrical instability of the heart or to plaque rupture. Low blood levels of EPA+DHA have been shown to present a risk for sudden cardiac death, and supplementation of the diet with as little as 840 mg/day of EPA+DHA has been proven effective against sudden cardiac death, while an intake of 2-4 g/day assists in reducing levels of pro-inflammatory factors in the blood that may predispose an individual to plaque rupture (16). As a preventive measure, the researchers recommend at least 2 servings of fish per week for a healthy population, or the equivalent intake of EPA+DHA through supplementation.

In a 2012 review, Mozaffarian and Wu noted that the beneficial effects of EPA and DHA, alone or in combination, on various pathways linked to cardiovascular health, warrant an increase in the intake of these long-chain fatty acids (17). They further noted that the reduction of platelet aggregation, improvement in cardiac diastolic filling and arterial compliance, and/or reduction in systemic inflammation, all of which are outcomes of ω-3 supplementation, result in a lower risk of atrial fibrillation, fatal cardiac events, and non-fatal cardiovascular events. They further recommend consumption of EPA

and DHA as either fish or fish oils, because of their well-documented beneficial effects on cardiovascular health. These findings corroborate the results of previous reviews, such as the publication by von Schacky and Harris (18). In a review article of both epidemiological and intervention studies, ω-3 fatty acid intake was linked to lower levels of CVD, lower blood pressure, heart rate, levels of inflammatory biomarkers and flow-mediated dilation (19). All of these are risk factors for the development of atherosclerosis.

As well as physical health, mental health of healthy individuals, as measured by the Short Form-12 (SF-12) mental health score, has been improved following ω-3 supplementation (20). This result is not surprising, given previous research linking lower plasma ω-3 fatty acid levels in patients with dementia than in people with normal cognitive function (21). In a study of children in the United Kingdom, those with a lower blood level of DHA exhibited poorer reading ability and poorer working memory performance, even after controlling for sex and socio-economic status (22). They also presented with increased oppositional behaviour, as rated by parents, and greater emotional liability. In an elderly Quebec population, those in moderately good health, as measured by both good physical condition and a lack of cognitive decline, exhibited higher plasma ω-3 fatty acid levels than their less healthy counterparts (23).

In a recent comprehensive review of the role of long-chain polyunsaturated fatty acids (LCPUFA) throughout the lifecycle, and of arachidonic acid (AA), EPA+DHA in particular, Jansson and Kiliaan (2014) noted the requirement of neuronal membranes for DHA and AA, particularly in infancy (24). A significant LCPUFA deficiency may lead to schizophrenia, Attention Deficit Hyperactive Disorder, and other neurological disorders. Supplementation with LCPUFA during the perinatal period may improve neural development, and subsequent improvement in cognition, in humans, not only in infancy but throughout life. Throughout the remainder of the lifecycle, the balance between ω-6 long chain fatty acids and their ω-3 counterparts is important in health maintenance. An ω-6:ω-3 ratio of 1:1 or 2:1 is recommended for optimal health, yet the average ratio in the North American population is estimated to be as much as 10:1 or even 20:1 (25). A 2014 study of the dietary habits of U.S. adults revealed that, in general, North Americans do not meet the recommended ω-3 fatty acid intake from foods alone (26). The authors suggest that supplementation may need to be considered, in order to help this population meet their daily needs. Similarly, both the Academy of Nutrition and Dietetics and the American Heart Association suggest a prudent approach in recommending an increased intake of ω-3 fatty acids (27, 10). Although both organisations recommend a preference for obtaining ω-3 fatty acids through fish consumption, both also recognise that supplementation may also be required in order to meet target values, particularly in at-risk populations.

1.2 Investigational Product

The investigational product, Ultimate Omega®, contains 1000mg fish oil (providing 225mg DHA, 325mg EPA and 90mg other fatty acids). The comparator product, contains 1000mg fish oil (providing 200mg DHA and 300mg EPA).

1.3 Risk/Benefits

1.3.1 Potential Risks

There are no known risks associated with the use of fish oil at the dosage proposed for this study. Health Canada's monograph for fish oil supports the use of up to 5000mg EPA + DHA in adults per day. Fish oil is known to reduce triglycerides and may increase LDL-C. Individuals using cholesterol-lowering medications or who have significant history of cardiovascular disease will be excluded from this study to mitigate this potential risk.

1.3.2 Known Potential Benefits

Depending on the dosage and ratio of EPA to DHA, fish oil supplementation is associated with several health benefits which include support for cardiovascular health, triglyceride reduction, support for cognitive function, and promotion of healthy mood balance. While theoretical benefits exist, the purpose of this clinical trial is not to investigate these benefits, rather it is to assess omega-3 fatty acids (specifically EPA and DHA) concentrations in the red blood cells after six (6) months of supplementation and serum phospholipid fatty acids after one (1) month of supplementation.

2 TRIAL OBJECTIVES AND PURPOSE

2.1 Primary Objectives

The primary objective of this clinical trial is to assess the relative efficacy of a 90% rTG Ultimate Omega® in comparison to another TG form of fish oil (Meg-3™, DSM Nutritional Products, Ltd.) Omega-3 by assessing the percentage of EPA and DHA in red blood cells after 6 months of supplementation and serum phospholipid fatty acid profile after 1 month of supplementation.

2.1 Secondary Objectives

Secondary objectives include the red blood cell fatty acid profile after 12, 16 and 24 weeks of supplementation and lipid profile (total cholesterol, LDL-C, HDL-C and triglycerides) after 6 months (24 weeks) of supplementation.

2.2 Additional Objectives

An additional objective of this clinical study is to assess the tolerability of the fish oil supplements over 6 months of supplementation.

3 TRIAL DESIGN

3.1 Overall Study Design

This will be a randomized, double-blind, parallel study design. Study participants will be randomized equally to one of two groups.

3.1.1 Study Centres

This study will be conducted at one (1) investigative site in Canada.

3.1.2 Study Duration

The duration of participant involvement will be approximately 28 weeks, based on a screening period of approximately 0-28 days, and a 24-week treatment period.

3.2 Study Flow Chart

Participants will visit the facility on seven (7) occasions, including a screening visit. The study flow chart is presented in **Figure 1**.

Figure 1 Study Flow Chart

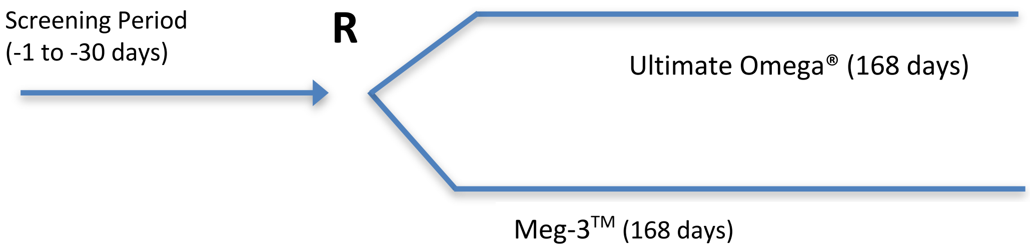

3.3 Schedule of Events

The schedule of events is presented in **Table 1**.

Table 1 Schedule of events by visit, including all procedures

Study Procedures							
**Study Visit**	**Pre-Screen**	**Screening (Visit 1)**	**Visit 2**	**Visit 3**	**Visit 4**	**Visit 5**	**Visit 6**
**Study Day**	**N/A**	**-1 to -30 days**	**Day 0**	**Day 28 ± 2**	**Day 84 ± 2**	**Day 112 ± 2**	**Day 168 ± 2**
**Telephone Pre-screening**	X						
**Participant Registration**		X					
**Informed Consent**		X					
**Physical Examination**		X					
**Review Medical History**		X	X				
**Review Medications**		X	X	X	X	X	X
**Demographic Data**		X					
**Vital Signs**		X	X	X	X	X	X
**Biometrics (height[1], weight, BMI)**		X	X	X	X	X	X
**Food Frequency Questionnaire**		X					
**Inclusion/Exclusion Criteria**		X	X				
**Fasting blood Sample (CBC, Glucose, AST, ALT, BUN, Creatinine, eGFR, Electrolytes [Na, K, Cl], Lipid Profile [Total cholesterol, HDL-C, LDL-C, Triglycerides])**		X					X
**Urine Pregnancy Test[2]**			X				X
**Randomization**			X				
**Blood Sample: Serum Phospholipid Fatty Acids**			X	X			
**Blood Sample: Total Red Blood Cell Fatty Acid Profile**			X		X	X	X
**Investigational Product Dispensed**			X	X	X	X	
**Investigational Product Returned**				X	X	X	X
**Treatment Diary Dispensed**			X	X	X	X	
**Treatment Diary Returned**				X	X	X	X
**Taste and Tolerability Questionnaire**				X	X	X	X
**Review Adverse Events**				X	X	X	X

[1] Height will be measured at visit 1 only
[2] Urine pregnancy test is for women of childbearing potential only

3.4 Narrative of Study Procedures

Prior to initiating any study-related procedures, all participants must provide written informed consent in accordance with Research Ethics Board (REB) requirements and applicable regulations.

Individuals who express an interest in participating in the study will be contacted, by phone, by a designated member of the research team. During the telephone call, the individual will answer specific questions in order to assess their eligibility for the study. If the participant meets the pre-screening requirements, the individual will be invited to schedule a screening visit no more than 2 to 30 days prior to the anticipated baseline (Day 0) visit with a study coordinator. Subjects will be asked to come to the screening visit fasting (nothing to eat or drink except water for at least 12 hours prior to the visit) and avoid consuming alcohol for at least 24 hours prior to the visit.

3.4.1 Visit 1 (-1 to -30 days prior to Day 0 – Screening Visit)

At this visit, the individual will meet with a study coordinator who will provide the individual with an informed consent form (ICF), which the individual will read and sign. Each participant will be given sufficient time to read the information carefully and ask the coordinator for more information. If necessary, the participant will also be given the option of taking the consent form home to review prior to making his/her decision to participate in the study.

Contraception: Women of childbearing potential (women who are not surgically sterile, or post-menopausal status) will be instructed to take the necessary precautions to avoid pregnancy for the duration of the study period. Women of childbearing potential must agree to one of the following acceptable contraceptive methods:

- Continuous use of oral or long acting injected contraceptive for at least 2 months prior to study entry , or
- Use of an intra-uterine device or implantable contraceptive, or
- Use of double barrier methods of birth control, or
- Abstinence from heterosexual intercourse

Women who are surgically sterile (or women with surgically sterile partners), and women who have achieved post-menopausal status (absence of menses for at least 12 months) will not be required to use contraceptive methods.

Once written informed consent is obtained from the participant, demographic and medical history data will be collected and a physical examination (excluding rectal / genital examination) will be performed. Biometrics (height and weight) and vital signs (seated resting blood pressure and heart rate) will be measured, and BMI calculated. Inclusion/exclusion criteria will be reviewed in order to confirm eligibility. If the participant does not meet the study requirements, the participant will be considered “screen failed”. Participants will be asked to complete a food frequency questionnaire to determine their omega-3 consumption habits. Changes in medical conditions and medications will be reviewed. Blood pressure, and weight will be measured and BMI calculated. A blood sample will be collected for analysis of CBC, glucose, AST, ALT, BUN, creatinine, eGFR, electrolytes (Na, K, Cl), and lipid profile (total cholesterol, LDL-C, HDL-C and triglycerides). Inclusion and exclusion will be reviewed in order to confirm eligibility. If the

participant does not meet the study requirements, the participant will be considered "screen failed". Eligible subjects will be scheduled for visit 2 and reminded to avoid consuming alcohol for at least 24 hours prior to visit 2.

3.1.1 Visit 2 (Day 0) [Baseline; Randomization]

Eligible subjects will return to the clinic for baseline assessments.

Participants who satisfy the study requirements will proceed with the following visit procedures:

- Urine pregnancy test (for women of childbearing potential only). If positive, individual will be deemed ineligible to participate, and will be considered "screen failed".
- Collection of vital signs (seated resting blood pressure and heart rate)
- Measurement of weight and calculation of BMI
- Review changes in medication / medical conditions
- Review of inclusion / exclusion criteria
- Blood sample: Serum phospholipid fatty acids and red blood cell fatty acids
- Randomization to treatment
- Administration of IP and IP dispensed
- Daily treatment diary will be dispensed

Subjects will be randomized to one of the following treatment regimens:

- Ultimate Omega® (5 capsules once per day in the morning with food) for 168 days
- Meg-3™ (5 capsules once per day in the morning with food) for 168 days

Subjects will be given detailed instructions by site personnel about the dosing regimen. Subjects will be instructed to take the first dose of the study product in the clinic with a snack. Subjects will remain in the clinic after taking the treatment for 15 minutes for observation.

Paper diaries will be provided to subjects for recording study product use, change in concomitant therapies, and any side effects/changes in current conditions.

Subjects will be reminded not to change dietary habits and activity/training level during the study and to avoid consuming alcohol for at least 24 hours prior to the next study visit.

The next visit will be scheduled for Day 28. In the event that this is not possible due to subject scheduling issues, a visit window of ±2 days may be applied. Subjects will be asked not to take their study product on the morning of their next visit.

3.1.2 Visit 3 (Day 28±2)

Subjects will return to the clinic on Day 28 (±2). Daily diaries will be collected and reviewed. All remaining study product and packaging will be returned. A new supply of study product will be dispensed.

At this visit, the following visit procedures will occur:

- Review changes in medication and adverse events
- Collection of vital signs (seated resting blood pressure and heart rate)

- Measurement of weight and calculation of BMI
- Calculation of investigational product compliance
- Taste and tolerability questionnaire
- Blood sample: Serum phospholipid fatty acids
- Administration of IP
- Daily treatment diary will be dispensed

Subjects will be given their daily dose of the study product in the clinic with a snack.

Paper diaries will be provided to subjects for recording study product use, change in concomitant therapies, and any side effects/changes in current conditions.

Subjects will be reminded not to change dietary habits and activity/training level during the study and to avoid consuming alcohol for at least 24 hours prior to the next study visit.

The next visit will be scheduled for Day 84. In the event that this is not possible due to subject scheduling issues, a visit window of ±2 days may be applied.

3.1.3 Visit 4 (Day 84±2)

Subjects will return to the clinic on Day 84 (±2). Daily diaries will be collected and reviewed. All remaining study product and packaging will be returned. A new supply of study product will be dispensed.

At this visit, the following visit procedures will occur:

- Review changes in medication and adverse events
- Collection of vital signs (seated resting blood pressure and heart rate)
- Measurement of weight and calculation of BMI
- Calculation of investigational product compliance
- Taste and tolerability questionnaire
- Blood sample: RBC fatty acid profile
- Administration of IP
- Daily treatment diary will be dispensed

Subjects will be given their daily dose of the study product in the clinic with a snack.

Paper diaries will be provided to subjects for recording study product use, change in concomitant therapies, and any side effects/changes in current conditions.

Subjects will be reminded not to change dietary habits and activity/training level during the study and to avoid consuming alcohol for at least 24 hours prior to the next study visit.

The next visit will be scheduled for Day 112. In the event that this is not possible due to subject scheduling issues, a visit window of ±2 days may be applied. Subjects will be asked not to take their study product on the morning of their next visit.

3.1.4 Visit 5 (Day 112±2)

Subjects will return to the clinic on Day 112 (±2). Daily diaries will be collected and reviewed. All remaining study product and packaging will be returned. A new supply of study product will be dispensed.

At this visit, the following visit procedures will occur:

- Review changes in medication and adverse events
- Collection of vital signs (seated resting blood pressure and heart rate)
- Measurement of weight and calculation of BMI
- Calculation of investigational product compliance
- Taste and tolerability questionnaire
- Blood sample: RBC fatty acid profile
- Administration of IP
- Daily treatment diary will be dispensed

Subjects will be given their daily dose of the study product in the clinic with a snack.

Paper diaries will be provided to subjects for recording study product use, change in concomitant therapies, and any side effects/changes in current conditions.

Subjects will be reminded not to change dietary habits and activity/training level during the study. Subjects will be asked to fast for at least 12 hours (nothing to eat or drink except water) prior to the visit and avoid consuming alcohol for at least 24 hours prior to visit 1.

The next visit will be scheduled for Day 168. In the event that this is not possible due to subject scheduling issues, a visit window of ±2 days may be applied. Subjects will be asked not to take their study product on the morning of their next visit.

3.1.5 Visit 6 (Day 168±2)

Subjects will return to the clinic on Day 168 (±2) for a final study visit. Daily diaries will be collected and reviewed. All remaining study product and packaging will be returned.

At this visit, the following visit procedures will occur:

- Review changes in medication and adverse events
- Collection of vital signs (seated resting blood pressure and heart rate)
- Measurement of weight and calculation of BMI
- Calculation of investigational product compliance
- Taste and tolerability questionnaire
- Urine pregnancy test (for women of childbearing potential only).
- Blood sample: CBC, AST, ALT, BUN, creatinine, eGFR, electrolytes (Na, K, Cl), and lipid profile (total cholesterol, LDL-C, HDL-C and triglycerides), RBC fatty acid profile

3.1.6 Unscheduled Visits

Any study participant who contacts the CRO about possible adverse events that are more than minor and/or that persist will be asked to visit the research centre for an assessment as appropriate.

3.1.7 Early Termination/Discontinuation Visit

Participants may withdraw voluntarily from participation in the study at any time. Subjects will be encouraged to visit the clinic for an early termination visit. At this visit, subjects will be encouraged to return study product and diaries, have blood pressure, heart rate and weight measured and provide a blood sample for analysis of safety parameters (hematology and clinical chemistry).

3.2 Diagnostic Tests

Subjects will be asked to fast for at least 12 hours (nothing to eat or drink except water) prior to the screening (Visit 1) and end of study visit (Visit 6). At these visits, blood samples will be collected by venipuncture for analysis of CBC, AST, ALT, BUN, Creatinine, eGFR, glucose, electrolytes (Na, K, Cl) and lipid profile (total cholesterol, LDL-C, HDL-C and triglycerides. Additionally, blood samples (no requirement for fasting) will be collected by venipuncture for analysis of total RBC fatty acids at visits 2, 4, 5 and 6, and serum phospholipids at visits 2 and 3.

The volume of blood to be collected is approximately 14mL at visit 1, 10mL at Visits 2 and 3, 5mL at visits 4 and 5 and 19mL at visit 6. The total amount of blood collected over the 6 month study period will be approximately 63mL.

3.3 Allocation to Treatment

Each participant will be randomized in a 1:1 to ratio to either the investigational product (Ultimate Omega®) or comparator product Meg-3™ as follows:

**Study Arm**	**Subject Number**
Ultimate Omega®	N=30 (15 Males and 15 Females)
Meg-3™	N=30 (15 Males and 15 Females)
**Total**	**60**

3.4 Stopping Rules/Discontinuation

The investigator may withdraw any participant from this research if circumstances arise that warrant doing so. If a participant is withdrawn from the study for any reason, they will be asked to complete an early termination visit.

3.5 Concomitant Medication(s)

3.5.1 Permitted Medication(s)

Prescription and OTC medications (including natural health products) not known to affect study endpoints are permitted if they have been consumed at a stable dose for the three months prior to Day 0.

Anti-hypertensive medications and thyroid hormone replacement therapy will be permitted provided subjects have been on a stable dosage (no change in dose) for the three months prior to screening (visit 1) and are not expecting to change dosage for the duration of the study.

Women of child-bearing potential will be permitted to continue using hormonal birth control.

3.5.2 Prohibited Medication(s)

Subjects will not be allowed to use the following medications during the study:

- Blood thinning agents (e.g. Warfarin)
- Corticosteroids
- Prescription anti-inflammatory medications
- Blood lipid-lowering medications or other lipid-binding medications (e.g. statins, fibrates, bile acid exchanger resin, phytosterols, niacin or its analogues, carnitine, supplements containing omega-3 fatty acids, polyglucosamines [Chitosan])
- Prescription or OTC medications (including NHPs) for weight loss or weight management

3.5.3 Washout Period

Subjects must not have used blood lipid-lowering medications or other lipid-binding medications, corticosteroids, anti-inflammatory medications or weight-loss programs or weight-loss medications in the 6 months prior to visit 1. Due to timing, it is unlikely that any subjects will be asked to wash out of the above medications or asked to stop diet programs in order to be eligible for this trial.

3.6 Measures to Minimize Bias

The investigational products will be similar in size and shape and will be filled in similar primary packaging to minimize bias. All investigational product will have similar labels, differing only in the randomization number in order to blind the products.

4 SELECTION AND WITHDRAWAL OF SUBJECTS

4.1 Number of Study Volunteers

Sixty (60) participants will be enrolled in this study, with equal numbers of male and female subjects assigned to each group.

Subjects will be recruited for this study from the Guelph, Ontario region located in Southwestern Ontario, Canada. Advertisement of the study will include using NDI's electronic subject database to identify potential candidates, e-mail and online advertisement, and posted paper advertisements. Participants interested in the study will contact the study recruiters via phone, e-mail or online. Following this first contact, the study will be discussed in more detail and additional information not found on the recruitment material will be provided. Following this conversation, potential participants will schedule a first study visit appointment. During this appointment, further questions or concerns about the study will be addressed and subjects will read the informed consent. Subjects will be given the opportunity to take the informed consent home to consider participating in the study and respond to the recruiter, or alternatively, subjects may sign and agree to participate in the study immediately. Once the informed consent is obtained, subjects will be screened against the inclusion/exclusion criteria to determine whether they may participate in the study.

4.2 Inclusion Criteria

An individual must meet all of the following inclusion criteria in order to be enrolled this study:

- I1. Male or female adults aged 18 – 35 (inclusive)
- I2. Healthy as determined from medical history
- I3. Non-smoker, or ex-smoker ≥6 months
- I4. Body mass index 18.5 – 24.9 kg/m^2
- I5. Female participants of childbearing potential (i.e. not surgically sterilized or post-menopausal greater than one year) must have negative urine pregnancy test and must be using an effective birth control method, defined as:
  - Continuous use of oral or long acting injected contraceptive for at least 2 months prior to study entry , or
  - Use of an intra-uterine device or implantable contraceptive, or
  - Use of double barrier methods of birth control, or
  - Abstinence from heterosexual intercourse
- I6. Willing to avoid alcohol consumption for 24 h prior to every clinic visit
- I7. Willing to maintain a stable body weight, activity level and dietary pattern except for use of the study products, as directed
- I8. Willing and able to provide informed written consent

4.3 Exclusion Criteria

A participant will be excluded from this study if any of the following criteria are met:

E1. Individuals taking prescription or non-prescription health products that may affect the study endpoint (e.g. corticosteroids, prescription anti-inflammatory drugs, blood lipid-lowering drugs (e.g. statins, fibrates, bile acid exchanger resin, phytosterols, niacin or its analogues, carnitine, etc.) in the previous 6 months

E2. Individuals taking any supplements with n-3 FAs, phytosterols, polyglucosamines (Chitosan) or other lipid-binding ingredients in the previous 6 months

E3. Individuals who consume omega-3 fatty acid rich fish (salmon, mackerel, herring) more than twice per month

E4. Unstable use (initiation or change in dose) of antihypertensive medications or thyroid hormone replacement medications within 3 months prior to visit 1

E5. Use of any weight-loss programs or weight-loss medications (prescription or over-the counter) including, but not limited to, lipase inhibitors, within 6 months prior to visit 1 and throughout the study

E6. Pregnancy or lactation, or participant unwilling to take appropriate contraceptives for the duration of the study

E7. History of blood clotting disorders or use of coagulation-inhibiting drugs (e.g. warfarin)

E8. Presence of major diseases such as diabetes, gastrointestinal, endocrine, cardiovascular, renal, or liver disease

E9. History of neurological disease (e.g. Parkinson's disease, stroke, traumatic brain injury, etc.)

E10. History of cancer (excluding non-melanoma skin cancer and basal cell carcinoma) in past 5 years

E11. Uncontrolled hypertension defined as a seated resting systolic blood pressure ≥140 mmHg and/or diastolic blood pressure ≥90 mmHg

E12. Abnormal laboratory test results of clinical significance, including, but not limited to, ALT or AST ≥1.5X the upper limit of normal at screening (visit 1)

E13. TG ≥400 mg/dL (≥4.52mmol/L) at screening (visit 1)

E14. Presence or history (past 6 months) of alcohol or drug abuse; alcohol use of >2 standard alcoholic drinks per day

E15. Participant has a known allergy or intolerance to fish/fish oil or any of the ingredients in the test products

E16. Participant is vegan

E17. Participant is unwilling or unable to abide by the requirements of the protocol

E18. Any condition that would interfere with the participant's ability to comply with study instructions, might confound the interpretation of the study, or put the participant at risk

E19. Participant has taken an investigational medicine or has participated in a research study within 30 days prior to the first study visit

4.4 Withdrawal Criteria

A participant may end his/her participation in a study at any time without consequence. If a participant withdraws from the study, the Investigator will make a reasonable effort to determine the reason for withdrawal from the study and record any adverse events experienced by the subject.

Personal reasons (participant initiated): As stated in the ICF, participants will be informed that their participation in the study is optional, and they can withdraw from the study at any time, without consequence or prejudice.

Clinical judgment of investigator/physician: A participant could also be withdrawn from the study if, in the opinion of the treating physician, it is not in the participant's best interest to continue (e.g. pregnancy, adverse events, need for prohibited medication, illness). In the event that a female participant becomes pregnant during the study, she will be withdrawn from the study immediately, and the pregnancy followed to resolution. Further, spontaneous abortion or any congenital anomaly/birth defect, in the case of a live birth, must be reported as an SAE to the REB and Health Canada within 15 days of becoming aware of the event. All serious adverse events will result in withdrawal from the trial.

Protocol violation: Participants who fail to comply with the protocol may be removed at the discretion of the investigator.

Subjects Participants who wish to withdraw from the study will be asked for their reason(s). Reason(s) for participant withdrawal will be recorded on the CRF for all participants who sign the informed consent form.

Reasons for withdrawal will be documented as one of the following:

- W.1 Participant requests withdrawal from the study
- W.2 Participant withdraws consent
- W.3 AE makes the continuation of the participant impossible or inadvisable
- W.4 Participant was incorrectly included in the study (discovered after enrollment not to have met the protocol entrance criteria)
- W.5 Participant refuses to comply with required study procedures
- W.6 Participant has confirmed pregnancy

5 INVESTIGATIONAL PRODUCT

5.1 Treatment Groups

The study will consist of two treatment groups. Participants will be randomly assigned, in equal proportions, to the two treatment groups. An equal number of male and female subjects will be randomized to each group. Alpha-numerical codes will be used to identify participants. At randomization each subject will receive a unique numerical randomization code.

5.2 Description of IP

The investigational product (IP) is Ultimate Omega® [NPN 80003338]

Manufacturer: Nordic Naturals Inc.

Formulation of the IP (per capsule):

Medicinal Ingredients: Fish Oil 1000mg (providing 325mg EPA, 225mg DHA and 90mg other Omega-3 Fatty Acids)

Non-medicinal ingredients: Gelatin, Glycerin, Natural Lemon Flavour, Rosemary extract, Water

5.1 Description of Comparator Product

The comparator product is Meg-3™ Formulation of the comparator product (per capsule):

Medicinal Ingredients: Fish Oil 1000mg (providing 300mg EPA and 200mg DHA)

Non-medicinal ingredients: Gelatin, Purified Water, Natural Lemon Flavour, Rosemary extract and Vitamin E

5.2 Investigational Product Packaging and Labeling

The IP and comparator products will be provided by the sponsor. Study products will be packaged in white HDPE bottles with a count of 150 capsules per bottle. The study products will be labeled with compliant labels. An example of a compliant label in Canada for a natural health product is provided below:

Protocol No. (No. de Protocole): 1007-008-PRO-040414 Randomization No. (No. de Randomisation): <insert>
Product name and lot number (Nom du Produit et No. de Lot):
Ultimate Omega® [NPN 80003338] Lot 141179 or Meg-3™ Lot 153143
Contains: 150 Capsules Expiry date (Date d'expiration):Jun 2017

Investigational Natural Health Product (Produit de santé naturel expérimentale)
Use under the supervision of a qualified investigator
(Pour être utilize sous la supervision d'un chercheur quilifié).

Sponsor (Promoteur) / Manufacturer (Promoteur & Fabricant):
Nordic Naturals
111 Jennings Drive
Watsonville, CA 95076

For investigational use only (Utilisation invesigational seulement). Keep out of reach of children (Tenir hors de portée des enfants). Store at room temperature (Conserver à température ambiante).
If any problems or questions (Si des problèmes ou des questions), telephone 855-378-4950.

Investigator Name: Dr. Anthony Bier Phone: 226-706-8906
Subject No.: ___________ Subject Initials:_________
Date dispensed:____________ Date returned:___________

5.3 Dosage and Administration of IP

Subjects will be instructed to take the investigational product as 5 capsules in the morning with food on a daily basis for 168 days.

5.4 Storage

All study products will be stored in a secure location at room temperature protected from heat and direct light. Only the Investigator or designated members of the research team will have access to the study products.

5.5 IP Accountability

NDI will maintain records pertaining to the IP received from the Sponsor, the date the IP was received, and the disposition of the IP (e.g., dispensing and use by study participants), or if unused, the return of the IP to the Sponsor or other mutually agreed upon disposition (e.g. destruction).

5.6 Blinding

The investigational product and comparator product will be similar in shape and size. Both products will be labelled in a similar manner and will only be differentiated by a randomization code to ensure the study is double-blind.

5.7 Unblinding

Unblinding should not occur except in the case of emergency situations. In the event that a serious adverse event occurs, for which the identity of the investigational product administered is necessary to manage the patient's condition, the treatment emergency code for that participant may be broken and the investigational product identified. The sponsor must be notified of any unblinding within 24 hours. Details of patients who are unblinded during the study will be included in the final report.

Unblinding of the data will not occur until after database lock to ensure integrity of the study data.

6 ASSESSMENT OF EFFICACY

All assessments of efficacy will be made on blood collected during the clinical study period.

6.1 Primary Endpoints

The primary endpoints are red blood cell EPA and DHA after 6 months (24 weeks) of supplementation and serum phospholipid fatty acid analysis after 1 month of supplementation.

6.2 Secondary Endpoints

Secondary outcomes include the red blood cell EPA and DHA after 12 and 16 weeks of supplementation and lipid profile (total cholesterol, LDL-C, HDL-C and triglycerides) after 6 months (24 weeks) of supplementation.

7 ASSESSMENT OF SAFETY

Adverse events will be reviewed at each study visit. Additionally, vital signs, body weight, BMI will be used as measures of product safety. Blood will be collected prior to and after 6 months of supplementation for analysis of safety parameters (hematology and clinical chemistry).

7.1 Adverse Events (AEs)

Health Canada's NNHPD define an adverse event as "any adverse occurrence in the health of a clinical trial subject who is administered an NHP that may or may not be caused by the administration of the NHP, and includes an adverse reaction, a serious adverse reaction and a serious unexpected adverse reaction". NNHPD defines an adverse reaction as "a noxious and unintended response to an NHP that occurs at any dose used or tested for the diagnosis, treatment or prevention of a disease or for modifying an organic function". Adverse events are further defined in ICH Guideline E2A that "an adverse event can therefore be any unfavorable and unintended sign (including an abnormal laboratory finding, for example), symptom or disease temporally associated with the use of a medicinal product, whether or not considered related to the medicinal product." Pre-existing conditions which worsen during a study are to be reported as AEs.

7.1.1 AE Reporting

AE reporting processes will be carried out in compliance with the protocol, the governing Institutional Review Board (IRB), and government regulations.

AEs are intended to be volunteered by subjects or observed by the investigator. All AEs are to be recorded on appropriate Case Report Forms (CRFs). During the study, subjects should record any adverse effects in their diary. At each visit the subject will be asked "Have you experienced any difficulties or problems since I saw you last"? Any adverse events (AEs) will be documented in the study record and will be classified according to the description, duration, intensity, frequency, and outcome. The Principal Investigator will assess any AEs and decide causality.

7.1.2 Unexpected Adverse Reaction

An unexpected adverse reaction is an adverse reaction, the nature and severity of which is not consistent with the applicable product information (e.g., Investigator's Brochure for an unapproved investigational product or package insert/summary of product characteristics for an approved product).

7.1.3 Plan for Grading Adverse Events:

The following 3-point scale will be used in grading the severity of adverse events noted during the study:

- Mild: Awareness of event but easily tolerated
- Moderate: Discomfort enough to cause some interference with usual activity
- Severe: Inability to carry out usual activity

7.1.4 Plan for Attribution of Adverse Events:

Adverse events will be monitored for each subject participating in the study and attributed to the study procedures / design by the principal investigator according to the following categories:

- Definite: An adverse event that followed a reasonable temporal sequence from administration of the study drug; followed a known response pattern to the study drug; when appropriate to the protocol, was confirmed by improvement after stopping the study drug (positive de-challenge) and by reappearance of the reaction after repeat exposure (positive re-challenge); and could not be reasonably explained by known characteristics of the subject's clinical state or by other therapies.
- Probable: An adverse event that followed a reasonable temporal sequence from administration of the study medication; followed a known response pattern to the study medication; when appropriate to the protocol, was confirmed by improvement after de-challenge; and could not be reasonably explained by the known characteristics of the subject's clinical state or by other therapies.

- Possible: An adverse event that followed a reasonable temporal sequence from administration of the study drug and followed a known response pattern to the study medication but could have been produced by the subject's clinical state or by other therapies. De-challenge information is lacking or unclear.

- Not related: An adverse event for which sufficient information existed to indicate that the etiology was unrelated to the study drug. Two or more of the following variables applied:
  a. The adverse event did not follow a reasonable temporal sequence after administration of the study drug.
  b. The adverse event was readily explained by the subject's clinical state or other therapies.
  c. Negative de-challenge – the adverse event did not abate upon dose reduction or cessation of therapy (assuming that it was reasonable to expect abatement of the adverse event within the observed interval).

7.1.5 Plan for Determining Seriousness of Adverse Events:

In addition to grading the adverse event, the principal investigator (or qualified designate) will determine whether the adverse event meets the criteria for a Serious Adverse Event (SAE). An adverse event is considered serious if it:

- is life-threatening OR
- results in in-patient hospitalization or prolongation of existing hospitalization OR
- results in persistent or significant disability or incapacity OR
- results in a congenital anomaly or birth defect OR
- results in death OR
- based upon appropriate medical judgment, may jeopardize the subject's health and may require medical or surgical intervention to prevent one of the other outcomes listed in this definition (Examples of such events are intensive treatment in an emergency room or at home for allergic bronchospasm; blood dyscrasias or convulsions that do not result in hospitalization; or the development of drug dependency or drug abuse) OR
- adversely affects the risk/benefit ratio of the study

An adverse event may be graded as severe but still not meet the criteria for a Serious Adverse Event. Similarly, an adverse event may be graded as moderate but still meet the criteria for an SAE. It is important for the principal investigator to consider the grade of the event as well as its "seriousness" when determining whether reporting to the REB is necessary.

7.1.6 Serious Adverse Event (SAE) Reporting

SAE reporting processes will be carried out in compliance with the protocol and the governing REB. Notification of any serious adverse events must be made in writing to the study sponsor within 24 hours of the Principal Investigator learning of the event (Scott Minton, sminton@nordicnaturals.com). All SAEs must also be recorded on an AE CRF. The REB will be notified by Nutrasource Diagnostics Inc. of all SAEs and unexpected adverse reactions as required by the governing REB.

Nutrasource Diagnostics Inc. will notify the therapeutic products directorate (TPD) via facsimile (613-941-2121) or electronic submission to the Canada Vigilance database of all serious adverse or unexpected adverse reactions on behalf of Nordic Naturals using the CIOMS form as follows:

a. If it is neither fatal nor life threatening, within 15 days after the day on which the sponsor becomes aware of the information; and
b. If it is fatal or life threatening, within seven days after the day on which the sponsor becomes aware of the information.
c. Where it is fatal or life-threatening, immediately where possible and, in any event, within 7 days after becoming aware of the information. Within 8 days after having informed Health Canada, submit as complete as possible, a report which includes an assessment of the importance and implication of any findings (if this was not already part of the initial report).

As part of the CIOMS form, a number unique to the AE will be generated. Any follow-up reports must contain this number followed by -02, -03, etc. for each follow-up report. If the blind was broken for the SAE, this must be recorded in the CRF, communicated to the Sponsor, and the identity of the investigational treatment included in the SAE report to Health Canada and the REB. If the blind is maintained, a follow-up report should be submitted to the Health Canada and the REB once the blind has been broken for the study. A record of successful transmission of SAE reports and follow-ups to the REB and Health Canada must be maintained in the Trial Master File (TMF).

An initial written report of any SAE will be submitted by the Investigator to the Sponsor within three business days of occurrence or notification of occurrence. Any necessary follow-up reports will be submitted within a reasonable time thereafter.

The follow-up report will give full details of the experience, including an assessment of the relationship to the test article(s) and will be sent to the Sponsor promptly. When contacting the Sponsor regarding SAEs, site personnel should be prepared to provide as much of the following information as is available at the time:

- Subject's initials and subject number
- Investigator's name and center number
- Protocol title and number
- Subject's date of birth, gender, and race
- Test article(s), date(s) of administration, if blinded, please indicate
- Concomitant medication(s): dose, route, duration of treatment, date of last dose
- Information regarding the AE:
    - Description
    - Dates the event began and ended

- Whether the experience resulted in death or was life-threatening
- Whether hospitalization was required or prolonged
- Any treatment(s) required
- Outcome(s) of treatment(s)
- Investigator's determination of relationship to IP

7.1.7 Follow-up of AEs

AEs, especially those for which the relationship to investigational product is suspected, should be followed up until they have returned to baseline status or stabilized.

If after follow-up, return to baseline status or stabilization cannot be established an explanation should be recorded in the study record.

7.2 Vital Signs

In office, seated resting blood pressure and heart rate will be determined from a single measurement. Blood pressure will be checked in both arms at the first examination. If a consistent interarm difference exists, the arm with the higher pressure will be used throughout the study. One arm will be chosen and used consistently throughout the study. The arm selected for use at the initial visit will be documented in the study file.

Prior to blood pressure measurements, subjects will be encouraged to use the restroom. The subject should be seated comfortably with the back supported and the upper arm bared without restrictive clothing. Feet should be flat on the floor, legs should not be crossed. The subject should rest in this position for at least 5 minutes prior to the reading and must not talk during the measurement.

The same recording method and the same equipment should be used for each subject throughout the study.

7.3 Biometrics

Height will be measured at baseline with shoes removed and back straight using a stadiometer. Height will be measured in centimeters to one decimal place (e.g. 182.3 cm). Height will only be measured at screening. Two separate measurements of height will be taken and averaged. If height varies more than 1 cm, a third measurement will be taken. The closest two measurements will be used for the final calculation of height.

Weight will be measured at every study visit using a beam scale. Two separate measurements of weight will be taken and averaged. If weights vary more than 0.5 kg, a third measurement will be taken. The closest two measurements will be used for final calculation of weight. Weight will be measured with subjects in light clothing.

Body mass index will be calculated as [weight (kg)] ÷ [height (m) squared] using the weight (kg) at each visit divided by the square of the height (m) measured at visit 1. BMI will be recorded to one decimal place with the units kg/m^2.

Measurements of height, weight and BMI will be rounded to the nearest tenth using standard rounding conventions (e.g. a value of 0.50 or greater [0.50 to 0.99] rounds up to the next whole number and anything less than 5 [0.49 to 0.00] rounds down). For example an average height of 182.35 cm will be rounded to 182.4 cm.

7.4 Hematology and Clinical Chemistry

Fasting (12 hours) blood samples will be collected at screening (Visit 1) and week 12 (Visit 6) at the end of the treatment period. Samples will be analyzed for complete blood count (CBC) with differential (hemoglobin, hematocrit, white blood cell count with differential, red blood cell count and platelet count), BUN, creatinine, eGFR, AST, ALT, and electrolytes (Na, K, Cl).

8 STATISTICS

Values of all primary and secondary variables will be summarized using descriptive statistics for all treatments. Safety analyses will include, where appropriate, descriptive statistics, counts and percentages.

8.1 Planned Interim Analysis

When approximately 100% of participants have completed Visit 3 (Day 28), an interim analysis to determine changes in serum phospholipid fatty acids will be performed by research staff not involved in the conduct of the clinical trial.

8.2 Sample Size

For this study, twenty four (24) subjects per group are expected to complete the study. With an expected attrition rate of 20%, thirty (30) subjects per group are required for this study. A formal sample size was not done for this investigative study.

8.3 Analytical Populations

8.3.1 Intent-To-Treat Population

Any subject known to start treatment (known to have taken ≥1 dose of study product) and completing at least one post-baseline visit will be included in the analysis of efficacy (intention-to-treat population).

8.3.2 Safety Population

Any subject known to start treatment (known to have taken ≥1 dose of study product) will be included in the analysis of safety.

8.4 Statistical Methods

An effectiveness analysis based on the intent-to-treat population will be performed for all efficacy outcomes. Continuous variables will be presented as means with standard errors of the mean (SEM). Categorical variables will be shown as frequencies or proportions. Numerical efficacy endpoints will be

formally tested for significance by analysis of covariance. The dependent variable will be the post-baseline value; the factor of interest will be the treatment group (product) and value at baseline (usually visit 2, except for some laboratory tests [e.g. lipid profile] where baseline is visit 1) will be the covariate.

The ANCOVA approach differs from the simpler approach of comparing changes from baseline between products in that the ANCOVA adjusts for baseline in a more sophisticated way than simply subtracting it from the end of study value. The adjustment allows the ANCOVA to compensate for regression to the mean, floor effects, and other ways that the baseline value could influence change from baseline.

Where values being summarized as significantly non-normally distributed, as assessed by the Anderson-Darling test, a non-parametric test, such as the Wilcoxon or Mann-Whitney U test will be used.

A within-group analysis, if warranted, may be performed using a Student’s paired samples t-test or Wilcoxon sign rank test.

All calculations and analyses will be performed using SAS version 9.2 or higher. For all statistical inferences, the tests will be performed at the two-sided, 0.05 level of significance.

8.5 Safety Analysis

Analysis of safety endpoints will be assessed for all subjects receiving medication (Safety Population). Safety analysis will be undertaken on all study participants (safety population).

Adverse events will be coded using the Medical dictionary for regulatory activities (MedDRA) terminology. Prior to statistical analysis, all AEs will be checked for currency of terms using the most current version of MedDRA. The version of MedDRA used for final coding of the adverse events will be documented in the final study report. AE data will be listed individually and summarized by body system and preferred terms within a body system for each treatment group. Serious and/or unexpected AEs will also be discussed on a case-by-case basis. For the tabulation of AEs by body system, a subject will be counted only once in a given body system. For example, a subject reporting 'nausea and diarrhea' will be reported as one subject, but the symptoms will be listed as two separate AEs within the class. Therefore, the total number of AEs reported within a body system may exceed the number of subjects within the body system reporting AEs.

Each AE (based on preferred terminology) will be only counted once for a given subject. If the same AE occurred on multiple occasions, the highest severity will be assumed. Thus, subjects are not counted multiple times in a given numerator in the calculation of reaction rates for a specific AE.

Statistical comparisons will be made using Fisher’s Exact test or Chi-Square test as appropriate.

Blood parameters (hematology and clinical chemistry) will be compared among groups using analysis of covariance using the screening value as the covariate. The dependent variable will be the post-baseline value; the factor of interest will be the treatment group (product) and value at screening (visit 1) will be the covariate.

8.6 Handling of Missing or Spurious Data

In the case of outliers or spurious data, that is, data that is not plausible, a rule of plus or minus 3 standard deviations of the mean will be applied to determine if the value should be excluded from analysis. All missing data or data excluded from analysis will be detailed in the final report. Missing values for primary and secondary endpoints at visits 3, 4, 5 or 6 will be imputed with the more recent previously-available value (LOCF, or "last-observation-carried-forward" imputation). No imputation will be performed for any missing daily diary variables, taste and tolerability questionnaire values or missing values of parameters of safety.

8.7 Premature Discontinuation Description

For each premature discontinuation, the following parameters will be listed in the final study report: subject number, dates of treatment start and end of treatment, and the reason for premature discontinuation.

8.1 Protocol Deviations and Violations

A log of protocol deviations and violations will be maintained during the course of the study. Deviations and violations will be reviewed by the investigator and impact on study data will be determined. All protocol deviations, violations and any data excluded from statistical analysis will be documented in the final study report.

8.2 Termination of the Trial

There are no specific stop criteria formulated for this study. In the case of complete premature termination of the trial, participating investigators/subjects, and the REB must be promptly informed of the termination and all study data and investigational product collected from study subjects.

9 QUALITY CONTROL AND QUALITY ASSURANCE

9.1 Auditing

All material used in clinical studies are subjected to quality control. Quality assurance audits may be performed by the sponsor or any health authority (e.g. Health Canada, FDA, etc.) during the course of the study or after its completion.

The Investigator agrees to comply with the sponsor and regulatory requirements in terms of auditing of the study. This includes access to the source documents for source data verification.

9.2 Monitoring

An initiation meeting will be conducted by the sponsor or an approved representative (CRO). At this meeting, the protocol and logistical aspects of the study will be reviewed with the Investigator and all study staff.

Nutrasource Diagnostics Inc. will assume the responsibility for monitoring the study on behalf of the Sponsor, Nordic Naturals. Monitoring activities may include review of source documents to ensure that all items have been completed and that the data provided are documented, accurate and obtained in the manner specified in the protocol. The subject files may be reviewed to confirm that:

- Informed consent was obtained and documented, signed by the participant, the Study Coordinator and the Principal Investigator
- Enrolled subjects fulfilled all inclusion criteria and did not meet any exclusion criteria;
- AE/SAE reporting has been performed as applicable;
- Study visits have been conducted as per protocol and information has been recorded in the appropriate place in the source document;
- The study product is being stored correctly and an accurate record of its dispensation to the study subjects is being maintained (accountability).

Incorrect, inappropriate, or illegible entries in the subject files will be returned to the Investigator or designee for correction. No data disclosing the identity of subjects will leave the study centre.

The Investigator and any designees will maintain confidentiality of all subject records.

The Investigator will permit trial-related monitoring, audits, REB review, and regulatory inspections and will allow direct access to source data and documents for these purposes.

10 ETHICAL CONSIDERATIONS

10.1 Ethical Principles

This study is to be conducted according to the protocol and international standards of Good Clinical Practice (International Conference on Harmonization guideline), applicable government regulations and Institutional research policies and procedures.

This protocol, a copy of the informed consent form, and, if applicable, subject recruitment materials and/or advertisements, and other documents required by applicable laws and regulations will be submitted to an REB for approval. The REB's written approval of the protocol and subject informed consent form must be obtained prior to commencement of this study. The REB approval must refer to the study by exact protocol title, number and version date; identify version of other documents (e.g., informed consent form) reviewed; and state the approval date. This study will be conducted under a protocol reviewed by an REB; the study is to be conducted by scientifically and medically qualified persons; the benefits of the study are in proportion to the risks; the rights and welfare of the subjects will be respected; the physicians conducting the study will ensure that the hazards do not outweigh the potential benefits; and the results to be reported will be accurate. Nutrasource Diagnostics Inc. must adhere to all requirements stipulated by the REB. This may include notification to the REB regarding protocol amendments, updates to the informed consent form, recruitment materials intended for viewing by subjects, local safety reporting requirements and submission of the investigator's annual/final status report to the REB.

10.1.1 Informed Consent

This study will be conducted in full compliance with the informed consent regulations (ICH E6). All subjects for this study will be provided a consent form describing this study and providing sufficient information for subjects to make an informed decision about their participation in this study. This consent form will be submitted with the protocol for review and approval by an REB(s). The Investigator, or designate, is responsible for obtaining the formal consent of a subject (competent to do so and not under duress), using the REB-approved Informed Consent Form (ICF); and this consent will be obtained before the subject undergoes any study procedure(s). This ICF must be signed by the subject or a legally acceptable representative, and the Investigator-designated study personnel obtaining the consent. A copy of the signed ICF will be given to the subject and the original retained by the investigator with the site's copy of the CRFs. The consent form must be reviewed and approved by the Sponsor prior to initiation of the study.

10.1.2 Research Ethics Board (REB)

This study will be conducted in full compliance with the REB regulations in ICH E6.

This protocol will not be initiated unless it has been reviewed and approved by, and remains open to continuing review by, an REB meeting the requirements of ICH E6. The REB shall review and have the authority to approve, require modification in (to secure approval), or disapprove the protocol. The REB shall notify the Investigator and the Institution in writing of its decision. The REB approval must refer to the study by exact protocol title, number and version date; identify version of other documents (e.g., informed consent form) reviewed; and state the approval date. The REB shall require that the information given to subjects as part of the informed consent is in accordance with ICH E6. The REB shall conduct continuing reviews of the protocol at intervals appropriate to the degree of risk, but not less than once per year. Copies of all reports to and correspondence between the Investigator and the REB must be provided to the Sponsor. Further, at the completion or early termination of the trial, a final report should be made to the REB by NDI.

It is the Investigator's obligation to maintain an REB correspondence file, and to make this file available for review by the Sponsor's representatives as part of the trial monitoring process.

10.2 Incentives and Benefits for the Subjects

There are no direct benefits from participating in this study. The risks associated with this study are low. If the study demonstrates efficacy, such as bioequivalence or superiority to the comparator product, Meg 3™, others could possibly benefit from using a high purity triglyceride form of fish oil, specifically Ultimate Omega®.

11 DATA HANDLING AND RECORD KEEPING

All data will be treated as confidential. Participants' identity/data will remain confidential through use of uniquely assigned ID code or number, but it will not be anonymous. Anonymous data cannot be traced back to an individual participant.

Participants will each be given a participant number, which will be the means of identifying blood samples and documents both during and after the clinical study. Only the principal investigator and designated

study staff will be privy to the identity names/numbers of the participants. Any findings released will be based on the collective results of the study, and no individual data will be revealed to anyone except the participant.

All records will be held under lockable conditions as per applicable regulatory requirements. Only designated members of the research team will have access to study records.

High safety standards for the transfer and storage of study data are guaranteed by the use of technologies such as password protection, firewalls and periodic backup to protect stored data. Access to study records and data will be limited to authorized personnel.

At critical junctures of the protocol (e.g., production of interim reports and final reports), data for analysis is locked and cleaned per established procedures.

An interim report will be provided to the Sponsor after approximately 100% of patients have completed Visit 3. A copy of the locked database and final study report will be provided to the Sponsor on completion of the trial.

All data is archived for a period not less than 25 years from the date of completion of the study in accordance with Health Canada regulatory requirements.

12 PROTOCOL AMENDMENTS AND END OF STUDY REPORT

12.1 Protocol Amendments

Once the protocol has been approved by the REB, any changes to the protocol must be documented in the form of an amendment. Alterations of the protocol may be made as the study progresses. Such changes will be captured in writing and will document the reasons for the change and must be signed and dated by the Sponsor. A detailed list of changes and rationale for changes will be appended to this protocol. Any such amendments may be subject to Health Canada and REB review/approval prior to implementation. All amendments will be documented in the final study report.

12.2 End of Study Report(s)

Once the study is completed, a study closure report will be filled out by the investigator and submitted to the REB. The end of the study is defined by the completion of all enrollment, interventions, data collections and analysis of identified data.

The Sponsor will be provided with a final study report on completion of the study and receipt of statistical analyses of all endpoints described in the protocol. The final study report will follow CONSORT and ICH guidelines for reporting study results.

13 DATA COLLECTION METHODS

All data will be collected/recorded on Case Report Forms (CRFs). The CRFs will be designed to capture evaluable data that is collected as a part of the study.

Case Report Forms will not include identifying information such as names, addresses or contact information.

14 FINANCING AND INSURANCE

All of the tests and study product, examinations, and medical care required as part of this study are provided at no cost to the subject, public health plan, or the subjects' private medical insurance (if any) and will be paid for by the study sponsor, Nordic Naturals. The subjects' public health plan, or the subjects' personal medical insurance (if any) should continue to pay for expenses for their current medical care and/or prescriptions. These expenses will not be paid as part of the subjects' participation in this study.

The sponsor, Nordic Naturals, is paying the investigator, NDI, for the time, effort and expenses to conduct this study. The study is fully financed by Nordic Naturals. Insurance coverage will be provided through NDI.

For participating and completing the study, subjects will be reimbursed. Should a participant not complete the entirety of the study due to withdrawal, disqualification or any other reason, reimbursement will be given based on the portion of the study completed. Parking will be covered for participants in the study.

In case of an injury or illness suffered by participation in this study, subjects will receive appropriate medical care. By signing the consent form, subjects do not give up their legal rights, nor release the study doctor or sponsors from their legal and professional obligations.

All results generated from the protocol will be owned by the sponsor, Nordic Naturals.

15 PUBLIC DISCLOSURE AND PUBLICATION POLICY

The research study will be registered by Nutrasource Diagnostics Inc. on behalf of the sponsor on Clinicaltrials.gov, a public registry and results database of clinical studies of human participants conducted around the world. Publication policy is covered under an agreement outside of this protocol.

16 REFERENCES

1. Schwalfenberg G (2006) Omega-3 fatty acids: their beneficial role in cardiovascular health. Canadian Family Physician 52: 734-740.

2. Wu JHY, Lemaitre RN, King IB, Song X, Sacks FM, Rimm EB, Heckbert SR, Siscovick DS, Mozaffarian D (2012) Association of plasma phospholipid long-chain omega-3 fatty acids with incident atrial fibrillation in older adults: the cardiovascular health study. Circulation 125: 1084-1093.

3. Oh R (2005) Practical applications of fish oil (ω-3 fatty acids) in primary care. J Am Board Fam Pract 18: 28-36.

4. Bucher HC, Hengstler p, Schindler C, Meier G (2002) N-3 polyunsaturated fatty acids in coronary heart disease: a meta-analysis of randomized controlled trials. Am J Med 112: 298-304.

5. Lopez LB, Kritz-silverstein D, Barrett-Connor E (2011) High dietary and plasma levels of the omega-three fatty acid docosahexaenoic acid are associated with decreased dementia risk: the Rancho Bernardo study. J Nutr, Health & Aging 15(1): 25-31.

6. Yurko-Mauro K, McCarthy D, Rom D, Nelson EB, Ryan AS, Blackwell A, Salem Jr. N, Stedman M, on behalf of the MIDAS Investigators (2010) Beneficial effects of docosahexaenoic acid on cognition in age-related cognitive decline. Alzheimer's and Dementia 6: 456-464.

7. Navqi AZ, Buettner C, Phillips RS, David RB, Mukamel KJ (2010) n-3 fatty acids and periodontitis in US adults. J Am Diet Assoc 110: 1669-1675.

8. Lee YH, Bae SC, Song GG (2012) Omega-3 polyunsaturated fatty acids and the treatment of rheumatoid arthritis: a meta-analysis. Arch Med Res 43(5): 356-62.

9. Dawczynski C, Hackermeier U, Viehweger M, Strange R, Springer M, Jahreis G (2011) Incorporation of n-3 pufa and γ-linolenic acid in blood lipids and red blood cell lipids together with their influence on disease activity in patients with chronic inflammatory arthritis--a randomized controlled human intervention trial. lipids health dis 10: 130-138.

10. http://www.heart.org/heartorg/gettinghealthy/nutritioncenter/healthydietgoals/fish-and-omega-3-fatty-acids_ucm_303248_article.jsp

11. Albert CM, Campos C, Stampfer MJ, Ridker PM, Manson JE, Willett, WC, Ma J (2002) Blood Levels of long-chain n–3 fatty acids and the risk of sudden death. New England Journal of Medicine 346 (15): 1113-1118.

12. Simon JA, Hodgkins ML, Browner WS, Neuhaus JM, Bernert JT, Hulley SB (1995) Serum fatty acids and the risk of coronary heart disease. American Journal of Epidemiology 142 (5): 469-476.

13. Lemaitre RN, King IB, Mozaffarian D, Kuller LH, Tracy RP, Siscovick DS (2003) N-3 Polyunsaturated fatty acids, fatal ischemic heart disease, and nonfatal myocardial infarction in older adults: the Cardiovascular Health Study. American Journal of Clinical Nutrition 77(2): 319-325.

14. Harris WS, von Schacky C (2004) The Omega-3 Index: a new risk factor for death from coronary heart disease? Preventive Medicine 39 (1): 212-220.

15. Shahar E, Folsom AR, Wu KK, Dennis BH, Shimakawa T, Conlan MG, Davis CE, Williams OD. (1993) Associations of fish intake and dietary n-3 polyunsaturated fatty acids with a hypocoagulable profile. The Atherosclerosis Risk in Communities (ARIC) Study Arterioscler Thromb. 13(8): 1205-12.

16. Rupp H, Wagner D, Rupp T, Schulte LM, Maisch B (2004) Risk stratification by the "EPA+DHA level" and the "EPA/AA ratio" focus on anti-inflammatory and antiarrhythmogenic effects of long-chain omega-3 fatty acids. Herz. 29(7): 673-85.

17. Mozaffarian D, Wu JH (2012) (n-3) fatty acids and cardiovascular health: are effects of EPA and DHA shared or complementary? J Nutr 142(3): 614S-625S.

18. von Schacky C, Harris WS (2007) Cardiovascular benefits of omega-3 fatty acids. von Schacky C, Harris WS. Cardiovasc Res. 73(2): 310-5.

19. Kelley DS, Adkins Y. (2012) Similarities and differences between the effects of EPA and DHA on markers of atherosclerosis in human subjects. Proc Nutr Soc. 71(2): 322-31.

20. Udani JK, Ritz BW. (2013) High potency fish oil supplement improves omega-3 fatty acid status in healthy adults: an open-label study using a web-based, virtual platform. Nutr J. 12(1): 112.

21. Cherubini A, Andres-Lacueva C, Martin A, Lauretani F, Iorio AD, Bartali B, Corsi A, Bandinelli S, Mattson MP, Ferrucci L. (2007) Low plasma N-3 fatty acids and dementia in older persons: the InCHIANTI study. J Gerontol A Biol Sci Med Sci. (10): 1120-6.

22. Montgomery P, Burton JR, Sewell RP, Spreckelsen TF, Richardson AJ. (2013) Low blood long chain omega-3 fatty acids in UK children are associated with poor cognitive performance and behavior: a cross-sectional analysis from the DOLAB study. PLoS One. 24; 8(6).

23. Fortier M, Tremblay-Mercier J, Plourde M, Chouinard-Watkins R, Vandal M, Pifferi F, Freemantle E, Cunnane SC. (2010) Higher plasma n-3 fatty acid status in the moderately healthy elderly in southern Québec: higher fish intake or aging-related change in n-3 fatty acid metabolism? 82(4-6): 277-80.

24. Janssen CI, Kiliaan AJ. (2014) Long-chain polyunsaturated fatty acids (LCPUFA) from genesis to senescence: the influence of LCPUFA on neural development, aging, and neurodegeneration. Janssen CI, Kiliaan AJ. Prog Lipid Res. 53: 1-17.

25. Simopoulos AP. (2002) The importance of the ratio of omega-6/omega-3 essential fatty acids. Biomed Pharmacother 56(8):365-79.

26. Papanikolaou Y, Brooks J, Reider C, Fulgoni VL 3rd. (2014) U.S. adults are not meeting recommended levels for fish and omega-3 fatty acid intake: results of an analysis using observational data from NHANES 2003-2008. Nutr J: 13:31.

27. Vannice G and Rasmussen H: (2014) Position of the academy of nutrition and dietetics: dietary fatty acids for healthy adults. J Acad Nutr Diet. 114(1): 136-53.
